# Supplementary material for: Preferential molecular recognition of heterochiral guests within a cyclophane receptor
Source: Nat Commun. 2023 Jan 16;14:243. doi: 10.1038/s41467-023-35851-3 (PMC9842753; doi:10.1038/s41467-023-35851-3)
Supplement: Supplementary file 1 — Supplementary Information [file 41467_2023_35851_MOESM1_ESM.pdf]

Preferential Molecular Recognition of Heterochiral Guests within a Cyclophane Receptor

Supplementary Information

Manuel Weh,[†] Kazutaka Shoyama,[‡] and Frank Würthner[*,†,‡]

[†]Institut für Organische Chemie, Universität Würzburg, Am Hubland, 97074 Würzburg, Germany

[‡]Center for Nanosystems Chemistry (CNC), Universität Würzburg, Theodor-Boveri-Weg, 97074 Würzburg, Germany

[*]E-mail: wuerthner@uni-wuerzburg.de

Supplementary Methods

Perylene bisimide *rac*-3a

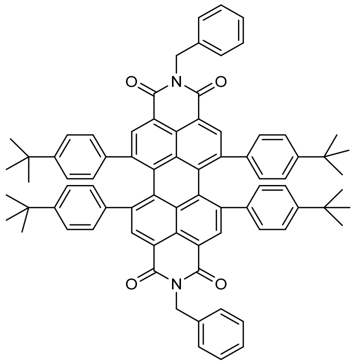

Racemic perylene bisanhydride *rac*-**2** (7.00 mg, 7.60 µmol, 1 eq.), benzylamine (500 mg, 4.67 mmol, 614 eq.) and imidazole (530 mg) were dissolved in dry toluene (5.0 mL) and stirred for 6 h at 120 °C under a nitrogen atmosphere. After being cooled down to room temperature, the solvent was removed under reduced pressure. The remaining solid was diluted with DCM and washed with 1 M HCl. The organic layer was dried over Na_2SO_4 and the solvent was removed under reduced pressure. The crude product was purified by preparative TLC (silica gel, DCM/cyclohexane 7:6), GPC (chloroform, 6.5 mL/min) and precipitation in DCM upon the addition of methanol. The supernatant was subsequently removed by centrifuging the mixture.

Yield: 8.30 mg (7.55 µmol, 99%) of a green solid.

HRMS (ESI, positive, acetonitrile/chloroform): (*m/z*) $[M]^+$, calcd. for $C_{78}H_{70}N_2NaO_4^+$: 1121.52333; found: 1121.51796.

^{1}H NMR (400 MHz, $CDCl_3$): δ = 8.30 (s, 4H), 7.64–7.62 (m, 4H), 7.39–7.28 (m, 6H), 7.04 (s, br, 8H), 6.58 (m, br, 8H), 5.54 (d, ^3J = 13.9 Hz, 2H), 5.33 (d, ^3J = 13.9 Hz, 2H), 1.36 (s, 36H).

^{13}C NMR (101 MHz, $CDCl_3$): δ = 164.0, 150.6, 142.1, 137.7, 137.5, 134.1, 132.2, 131.5, 129.4, 128.7, 128.2, 127.8, 126.0, 122.1, 43.8, 34.7, 31.4.

m.p.: >300 °C.

Perylene bisimide *rac*-3b

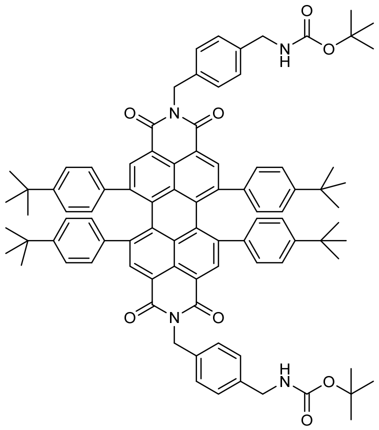

Racemic perylene bisanhydride **2** (141 mg, 153 µmol, 1 eq.), *N*-boc-4-(aminomethyl)-benzylamine (164 mg, 694 µmol, 4.5 eq.) and imidazole (2.20 g) were dissolved in dry toluene (140 mL) and stirred for 5.5 h at 120 °C under a nitrogen atmosphere. After being cooled down to room temperature, the solvent was removed under reduced pressure, the remaining solid was diluted with DCM and washed with 1 M HCl. The organic layer was dried over Na_2SO_4 and the solvent removed under reduced pressure. The crude product was purified by column chromatography (silica gel, DCM → DCM, 2% MeOH).

Yield: 189 mg (139 µmol, 91%) of a green solid.

HRMS (ESI, positive, acetonitrile/chloroform): (*m/z*) $[M]^+$, calcd. for $C_{90}H_{92}N_4O_8^+$: 1356.69152; found: 1356.68400.

^{1}H NMR (400 MHz, $C_2D_2Cl_4$): δ = 8.28 (s, 4H), 7.56 (d, ^3J = 8.3 Hz, 4H), 7.29 (d, ^3J = 8.3 Hz, 4H), 7.05 (d, br, ^3J = 11.2 Hz, 8H), 6.70 (s, br, 4H), 6.49 (s, br, 4H), 5.54 (d, ^3J = 14.0 Hz, 2H), 5.27 (d, ^3J = 14.0 Hz, 2H), 4.95 (t, ^3J = 5.4 Hz, 2H), 4.31 (d, ^3J = 5.9 Hz, 4H), 1.44 (s, 18H), 1.36 (s, 36H).

^{13}C NMR (101 MHz, $CDCl_3$): δ = 164.0, 156.0, 150.6, 142.1, 138.6, 137.7, 136.6, 134.1, 132.2, 131.5, 129.7, 128.2, 127.8, 126.0, 122.0, 44.5, 43.5, 34.7, 31.4, 28.5.

m.p.: 136–138 °C (decomposition).

Racemate of perylene bisimide cyclophanes 1-*MM* and 1-*PP*

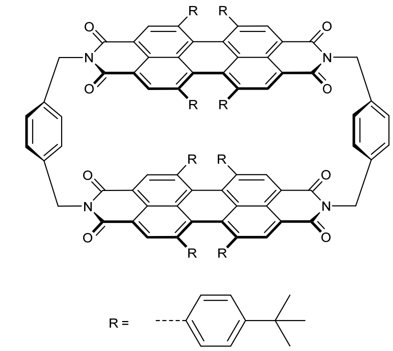

Perylene bisimide *rac*-**3b** (200 mg, 147 µmol, 1 eq.) was dissolved in DCM (30 mL). Subsequently, TFA (6 mL) was added and the mixture was stirred for 1 h at r.t.. The solvent was removed under reduced pressure and the residual solid was dissolved in DCM again. Subsequently, the solvent was again removed under reduced pressure in order to fully eliminate the remaining TFA. Next, racemic perylene bisanhydride **3** (136 mg, 148 µmol, 1 eq.) and imidazole (3.80 g) were added, the resulting mixture was dissolved in dry toluene (650 mL) and stirred for 28 h at 120 °C under a nitrogen atmosphere. After being cooled down to r.t., the solvent was removed under reduced pressure, the remaining solid was diluted with DCM and afterwards washed with 1 M HCl. The organic layer was dried over Na_2SO_4 and the solvent was removed under reduced pressure. The crude product was purified by column filtration (silica gel, DCM, 5% MeOH) and by GPC (chloroform, 6.5 mL/min). The pure compound was precipitated from chloroform upon the addition of methanol and the supernatant removed by centrifuging the mixture.

Yield: 60.0 mg (29.4 µmol, 20 %) of a green solid.

HRMS (ESI, positive, acetonitrile/chloroform): (*m/z*) $[M+Na]^+$, calcd. for $C_{144}H_{128}N_4NaO_8^+$: 2063.96244; found: 2063.97050.

^{1}H NMR (400 MHz, $CDCl_3$): δ = 8.18 (s, 4H), 7.79 (s, 4H), 7.55 (s, 8H), 6.92 (s, br, 8H), 6.74 (dd, ^3J = 8.1 Hz, ^4J = 1.9 Hz, 4H), 6.64 (dd, ^3J = 8.1 Hz, ^4J = 1.9 Hz, 8H, overlapping with broad signal), 6.40 (s, br, 4H), 5.85 (dd, ^3J = 8.1 Hz, ^4J = 2.0 Hz, 4H), 5.65–5.61 (m, 8H), 5.18 (d, ^2J = 13.4 Hz, 4H), 1.36 (s, 36H), 1.28 (s, 36H).

^{13}C NMR (101 MHz, $CDCl_3$): δ = 164.0, 163.5, 150.8, 149.2, 142.0, 141.5, 137.5, 137.1, 136.5, 134.3, 132.4, 132.3, 131.4, 131.1, 130.6, 127.9, 127.6, 127.3, 125.6, 124.1, 122.4, 121.6, 42.8, 34.61, 34.58, 31.46, 31.3.

m.p.: >300 °C.

UV/vis ($CHCl_3$, c_T = 30 µM, nm): λ_{max} (ε_{max}, M^{-1} cm^{-1}) = 304 (77 × 10^3), 477 (33 × 10^3), 620 (31 × 10^3).
Fluorescence ($CHCl_3$, nm): λ_{max} (λ_{ex}) = 705 (580).

The resulting enantiomers were separated by chiral HPLC (Reprosil, DCM)
1-*PP*: Retention time (Trentec, Reprosil 100 chiral-NR, *Ø* = 0.8 cm, DCM, flow: 1.0 mL/min): 3.2 min; λ_{max} ($\Delta\varepsilon$): 304 nm (+ 81 M^{-1} cm^{-1}). **1-*MM***: Retention time (Trentec, Reprosil 100 chiral-NR, *Ø* = 0.8 cm, DCM, flow: 1.0 mL/min): 4.3 min; λ_{max} ($\Delta\varepsilon$): 304 nm (– 90 M^{-1} cm^{-1}). The stereochemical assignment of the isolated diastereomers was achieved by comparison of the CD spectra with those of the previously reported enantiomerically pure *P*- and *M*-configured bay-tetraphenyl-substituted PBIs.[1]

1-*PP*: **CD** ($CHCl_3$, nm): λ_{max} ($\Delta\varepsilon_{max}$, M^{-1} cm^{-1}): 304 (+ 199), 334 (+ 277), 375 (+ 62), 459 (+ 31), 619 (+ 69).
1-*MM*: **CD** ($CHCl_3$, nm): λ_{max} ($\Delta\varepsilon_{max}$, M^{-1} cm^{-1}): 304 (– 200), 334 (– 274), 375 (– 67), 459 (– 32), 619 (– 67).

Perylene bisimide 18

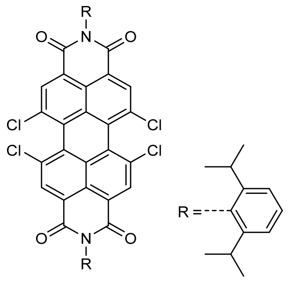

Perylene bisimide **18** was synthesized according to a literature known procedure:[2] Commercially available 1,6,7,12-tetrachloroperylene-3,4:9,10-tetracarboxylic acid (6.0 g, 11.3 mmol) and 2,6-diisopropylaniline (8.03 g, 45.3 mmol) were mixed in propionic acid (130 mL) and stirred for 19 h at 130 °C. The reaction was cooled down to room temperature. The solid was filtered and subsequently washed with aqueous $NaHCO_3$ and water and then dried under reduced pressure.
Yield: 5.5 g (6.5 mol, 57%) of a red solid.
The analytical data was in accordance with literature.[2]

Perylene bisimide *rac*-19

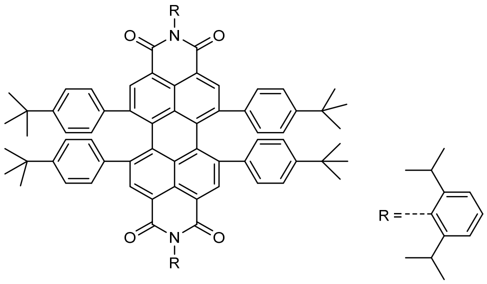

Perylene bisimide **19** was synthesized according to a literature known procedure:[3] A Schlenk-tube was charged with tetrachloro PBI **18** (2.50 g, 3.0 mmol), *para*-*tert*-butylphenylboronic acid (10.5 g, 58.9 mmol), tetrakis(triphenylphosphine)palladium(0) $[Pd(PPh_3)_4]$ (1.02 g, 0.88 mmol), K_2CO_3 (4.27 g, 30.9 mmol) under an inert atmosphere. Subsequently, 50 mL toluene, 10 mL ethanol and 25 mL water was added to the mixture. All solvents were independently degassed prior to use. Then, freeze-pump thaw technique was applied to further degas the mixture in three cycles. Subsequently, the reaction mixture was heated to 80 °C for 4 days. After cooling down to room temperature, the reaction mixture was extracted with dichloromethane. The combined organic phases were dried over $MgSO_4$ and the solvent was removed under reduced pressure. The crude product was purified by column chromatography (gradient of dichloromethane/cyclohexane 1:2 to 1:1).

Yield: 2.42 g (1.95 mmol, 66%) of a green solid.

The analytical data was in accordance with literature.[3]

Perylene bisanhydride *rac*-2

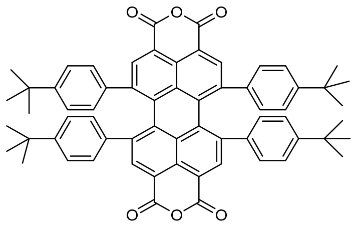

Perylene bisanhydride **2** was synthesized according to a literature known procedure:[3] Under an inert atmosphere **19** (2.21 mg, 1.8 mmol) was suspended together with KOH (100 g, 1.78 mol) in a mixture of *tert*-butanol (50 mL) and water (5 mL) and subsequently heated to 95 °C for 16 h. After cooling down to room temperature, the reaction mixture was poured into 500 mL cold 10% aqueous HCl and the resulting precipitate was collected and washed with water. The crude product was dissolved in dichloromethane, washed with water and the solvent was

removed under reduced pressure. The pure product was obtained by column chromatography (gradient of cyclohexane/dichloromethane 1:1 to 1:4.

Yield: 976 mg (1.05 mmol, 59%) of a green solid.

The analytical data was in accordance with literature.[3]

***tert*-butyl 4-(aminomethyl)benzylcarbamate (20)**

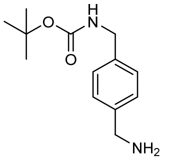

<u>Compound **20** was synthesized according to a literature known procedure</u>:[4] *p*-xylylenediamine (500.0 mg, 3.67 mmol) and triethylamine (TEA, 0.5 mL) were dissolved in 150 mL chloroform. Then $(Boc)_2O$ (800.0 mg, 3.67 mmol) was dropwise added to the solution at r.t. The reaction mixture was stirred overnight. Afterwards, the solvent was removed under reduced pressure, and the crude product was purified by dry load column chromatography (silica gel, eluent CH_2Cl_2, and then CH_2Cl_2/MeOH 20:1).

Yield: 35 % (303.5 mg, 1.30 mmol) of a colorless solid.

The analytical data was in accordance with literature.[4]

(Naphthalen-2-ylmethyl)triphenylphosphonium bromide (21)

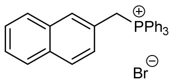

<u>Compound **21** was synthesized according to a literature known procedure</u>:[5] Commercially availbable 2-(bromomethyl)naphthalene (15.2 g, 68.7 mmol) and triphenylphosphine (18.1 g, 69.0 mmol) were mixed in *o*-xylene (350 mL) and heated to 160 °C for 2 h. The mixture was cooled down to room temperature and the solid filtered and washed with diethylether. The product was obtained pure after being dried under vacuum.

Yield: 31.7 g (65.6 mmol, 95%) of a white powder.

The analytical data was in accordance with literature.[5]

(*Z*)-2-[2-(Naphth-2-yl)vinyl]naphthalene (22)

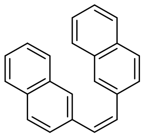

Compound **22** was synthesized in the style of a literature known procedure:[6] 2-Napthaldehyde (1.65 g, 10.6 mmol), (naphthalen-2-ylmethyl)triphenylphosphonium bromide (**21**) (5.15 g, 10.7 mmol) and LiOH·H_2O (1.40 g) were dissolved in *i*PrOH (200 mL) and stirred at 100 °C for 4 h. The mixture was cooled down to r.t. before the solvent was removed under reduced pressure. The crude product was again dissolved in DCM and washed with water. The organic phase was separated and the solvent was removed under reduced pressure. Purification by column chromatography (toluene) gave access to **22**.

Yield: 109 mg (389 µmol, 4%) of a white solid (several mixed fractions including the *E*-isomer were additionally obtained).

The analytical data was in accordance with literature.[6]

[5]Helicene (17)

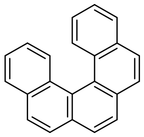

Compound **17** was synthesized in the style of a literature known procedure:[7,8] (*Z*)-2-[2-(naphth-2-yl)vinyl]naphthalene (**22**) (109 mg, 389 µmol) was dissolved together with I_2 (32 mg, 126 µmol) and thf (10 mL) in cyclohexane (300 mL) and stirred under irradiation (254–575 nm) for 1 h. Aqueous $Na_2S_2O_3$ was added to quench I_2. The phases were separated and the organic solvent was removed under reduced pressure. The desired product was obtained after column chromatography (cyclohexane/DCM 9:1) and GPC (chloroform, 6.5 mL/min).

Yield: 22 mg (79.0 µmol, 20%) of a white powder.

The analytical data was in accordance with literature.[7]

Supplementary Figures

a)

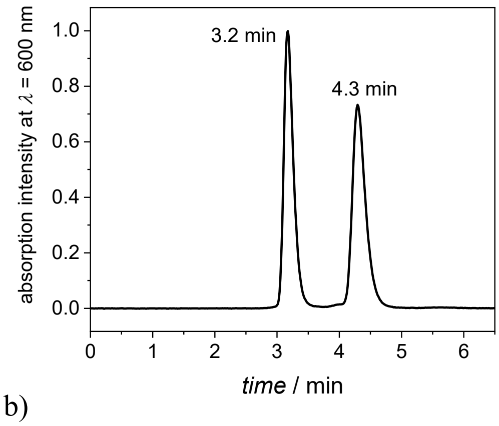

b)

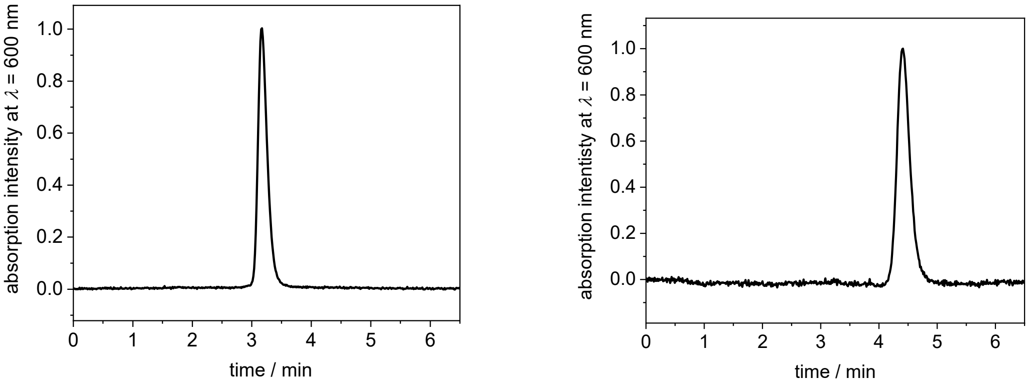

Supplementary Fig. 1 Analytical HPLC chromatogram a) for the separation of **1-*MM*** and **1-*PP*** and b) of the corresponding enantiomerically pure compounds which were separated by semipreparative chiral HPLC (DCM, flow: 1 mL/min).

Supplementary Note 1

The enantiomeric excess (*ee*) values were calculated by equation S1, where *a(peak1)* and *a(peak2)* refer to the integrals of the peaks for the first and second eluted isomer, respectively. For both enantiomers, *ee* values of >99% could be determined.

$$ee = \frac{a(peak\ 1) - a(peak2)}{a(peak\ 1) + a(peak2)} \times 100\% \qquad \text{(S1)}$$

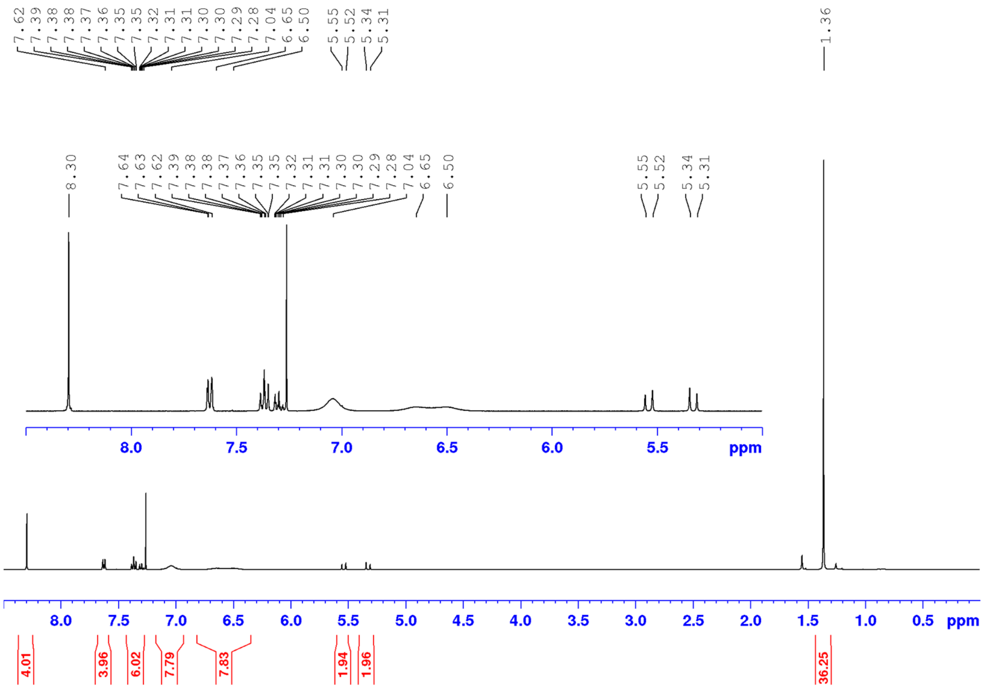

Supplementary Fig. 2 ^{1}H NMR (400 MHz) spectrum of compound *rac*-**3a** in $CDCl_3$ at 295 K.

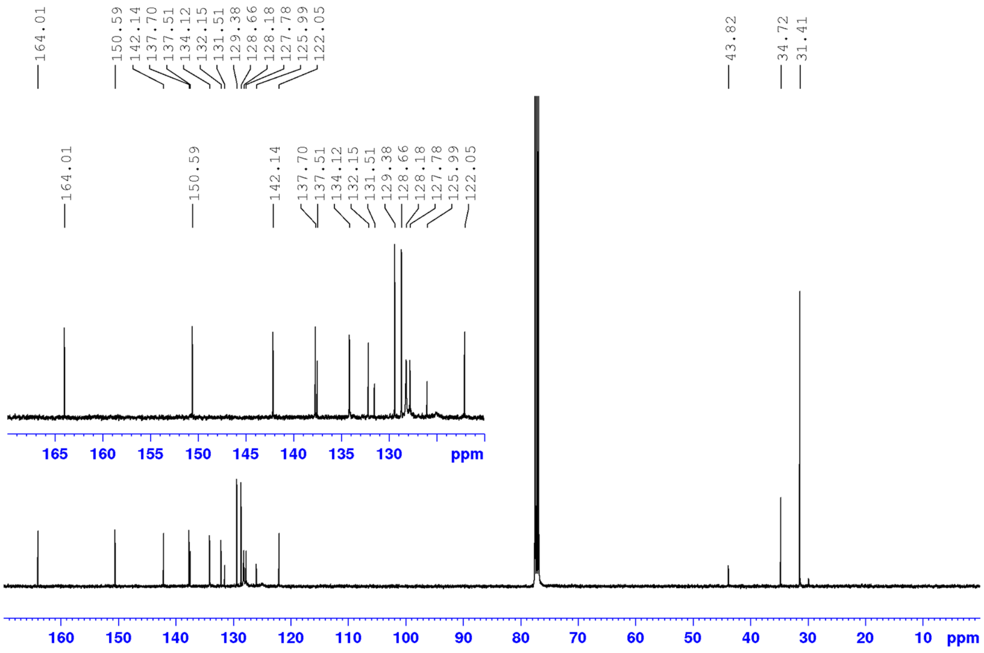

Supplementary Fig. 3 ^{13}C NMR (101 MHz) spectrum of compound *rac*-**3a** in $CDCl_3$ at 295 K.

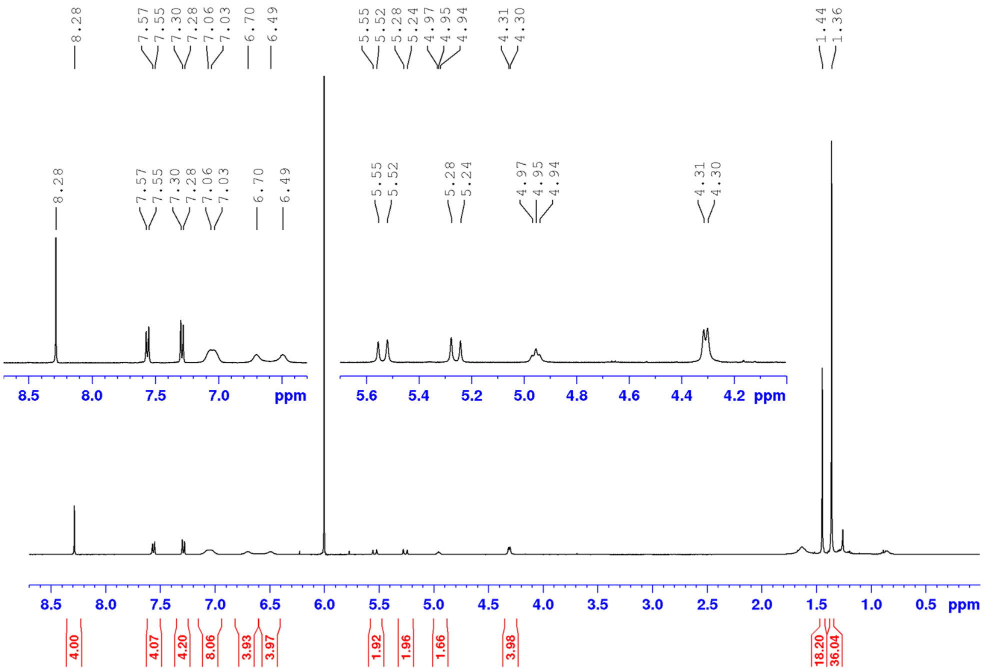

Supplementary Fig. 4 ^{1}H NMR (400 MHz) spectrum of compound *rac*-**3b** in $C_2D_2Cl_4$ at 295 K.

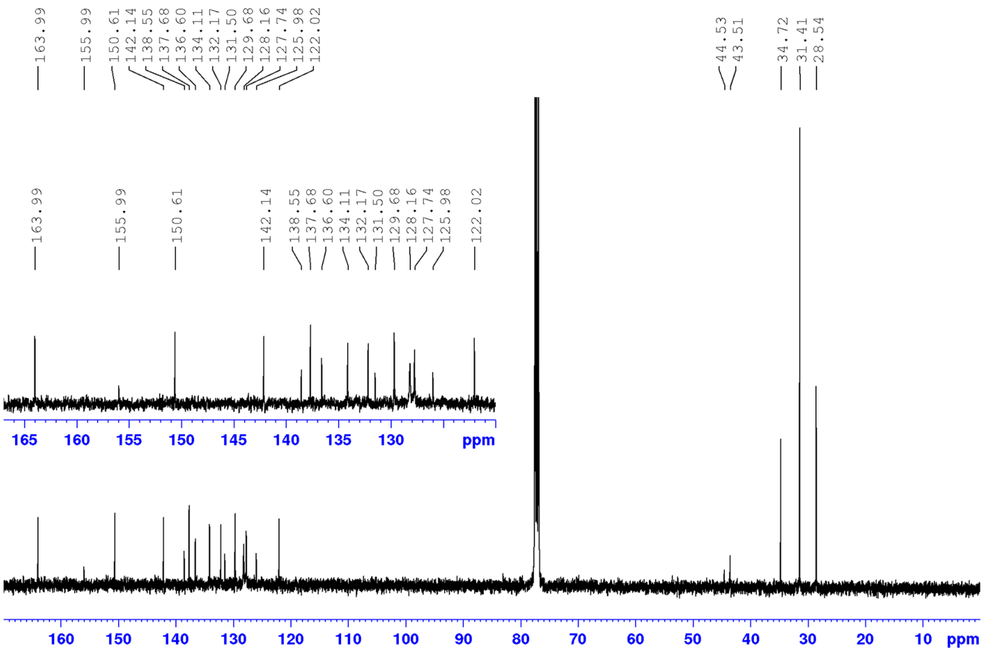

Supplementary Fig. 5 ^{13}C NMR (101 MHz) spectrum of compound *rac*-**3b** in $CDCl_3$ at 295 K.

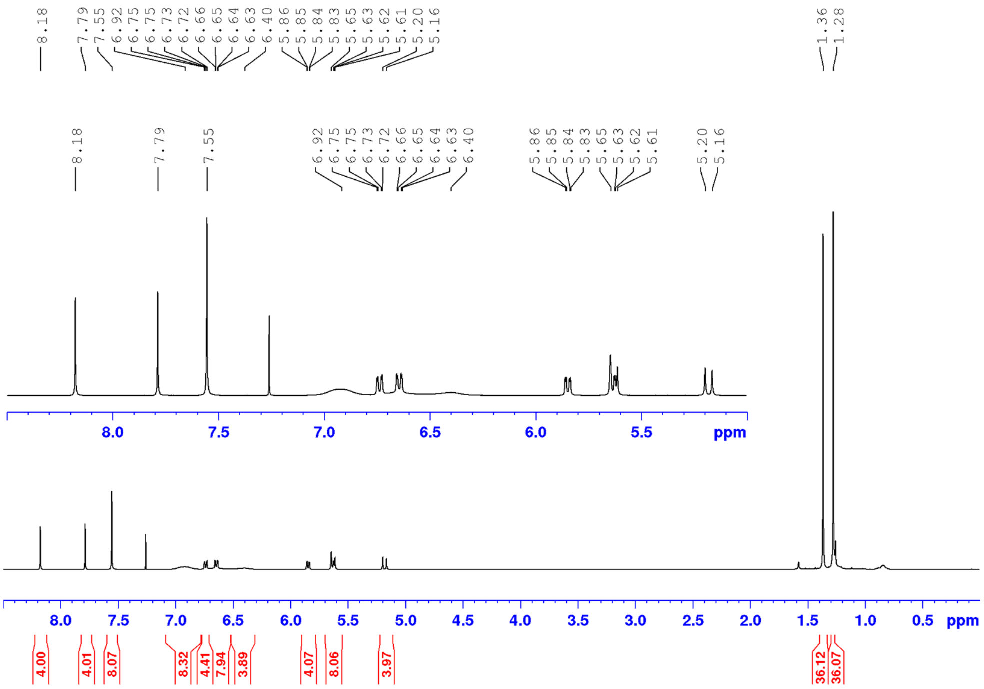

Supplementary Fig. 6 ^{1}H NMR (400 MHz) spectrum of compound *rac*-**1** in $CDCl_3$ at 295 K.

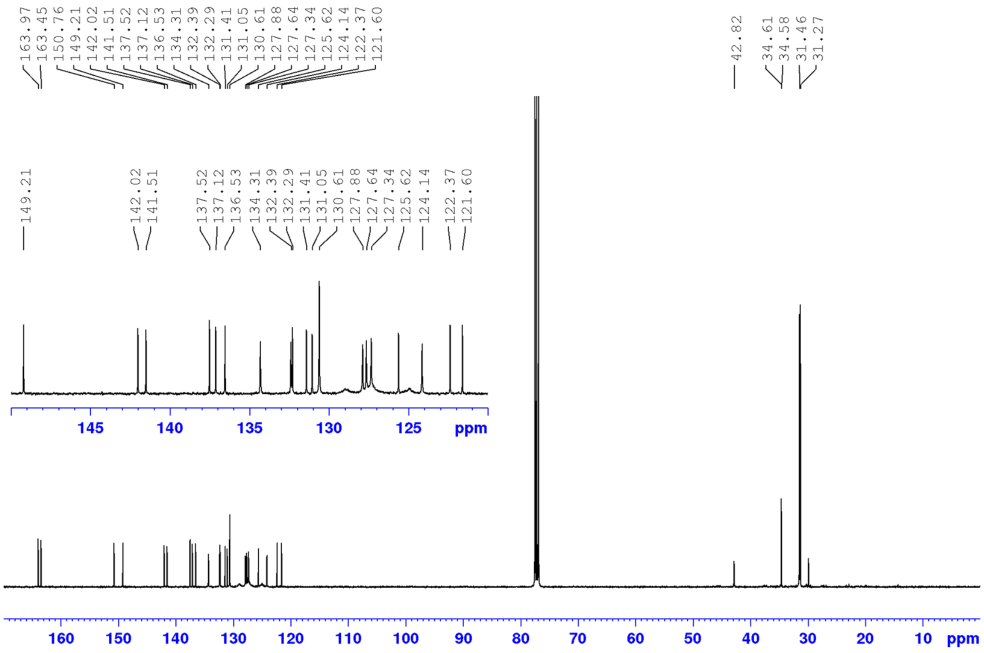

Supplementary Fig. 7 ^{13}C NMR (101 MHz) spectrum of compound *rac*-**1** in $CDCl_3$ at 295 K.

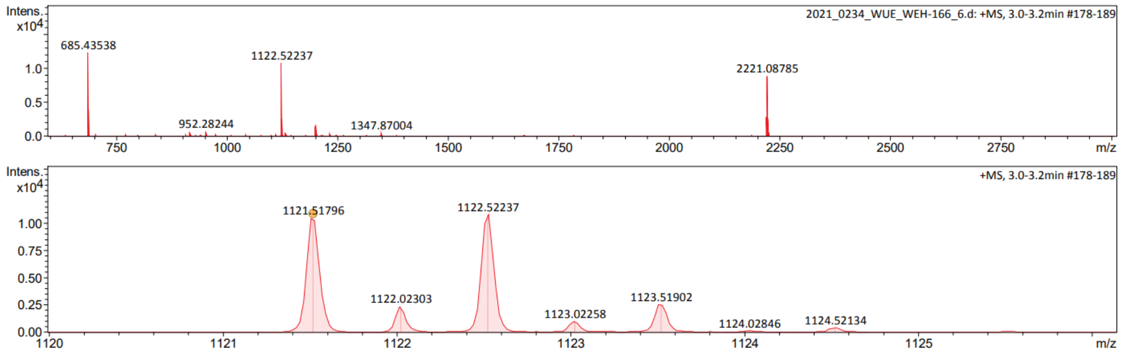

Supplementary Fig. 8 HRMS (ESI, positive, acetonitrile/chloroform) of compound *rac*-**3a**.

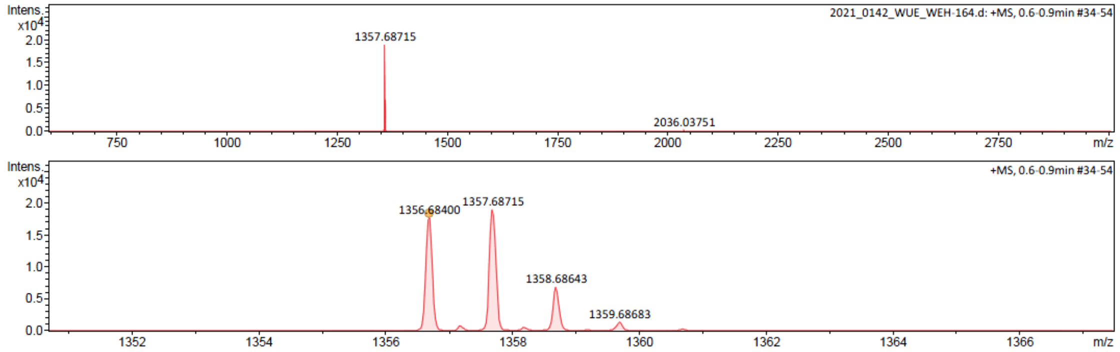

Supplementary Fig. 9 HRMS (ESI, positive, acetonitrile/chloroform) of compound *rac*-**3b**.

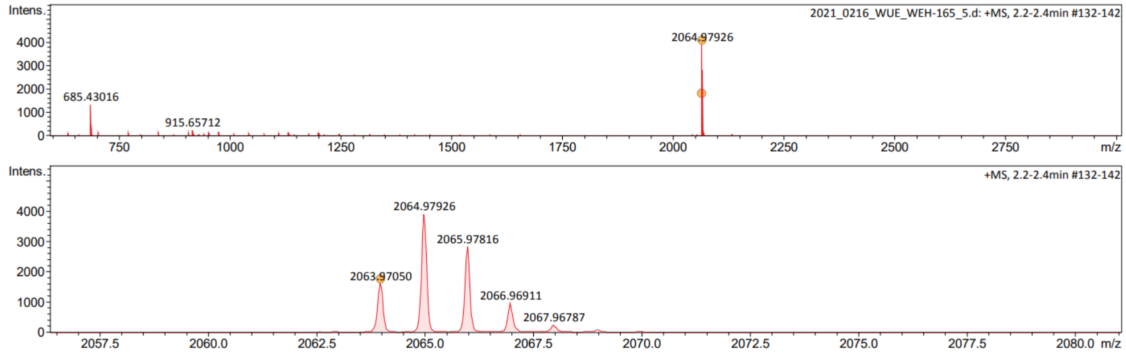

Supplementary Fig. 10 HRMS (ESI, positive, acetonitrile/chloroform) of compound *rac*-**1**.

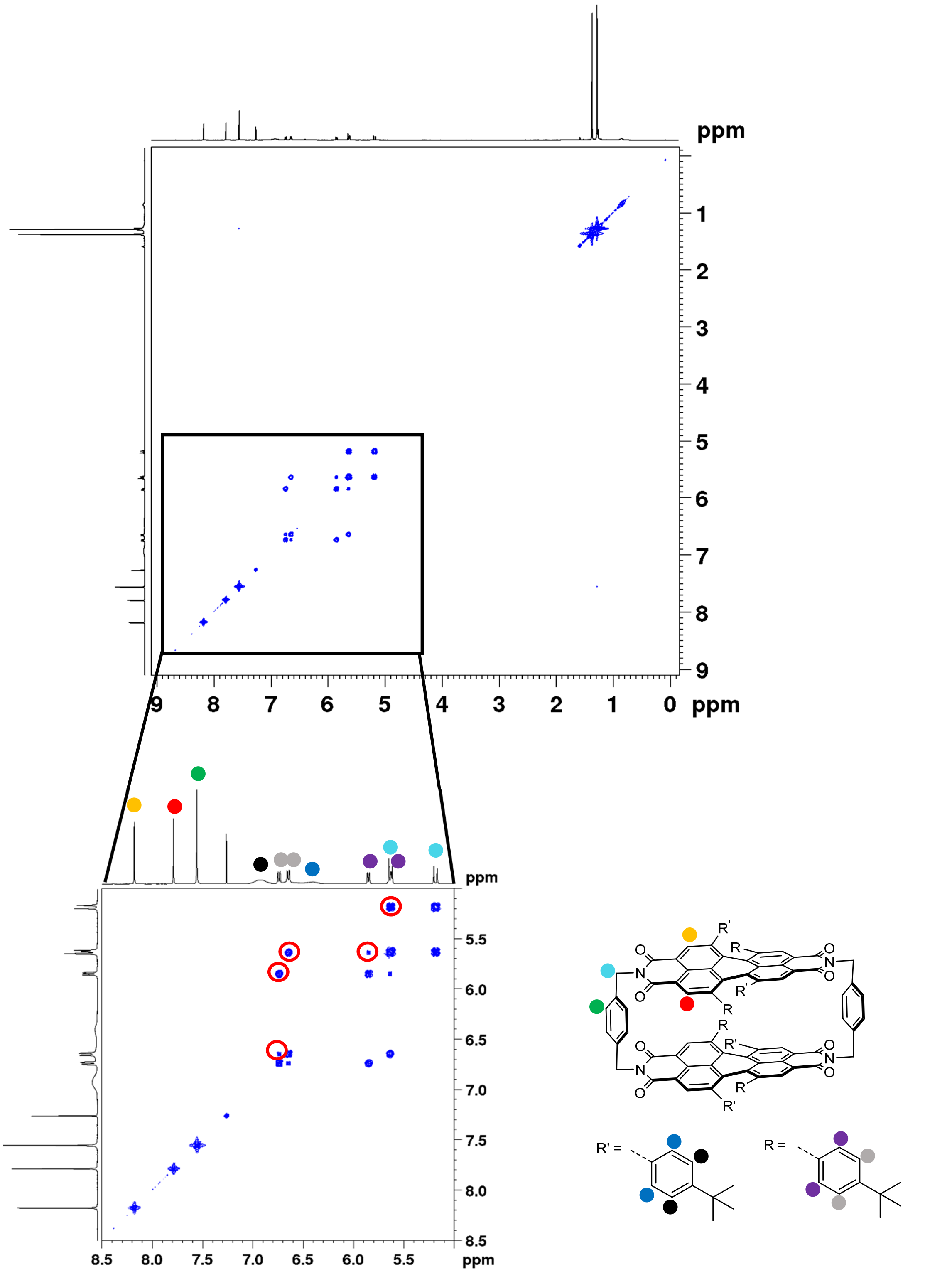

Supplementary Fig. 11 ¹H-¹H COSY NMR spectrum (400 MHz, 295 K) of *rac*-**1** in $CDCl_3$. The important cross signals are marked in red.

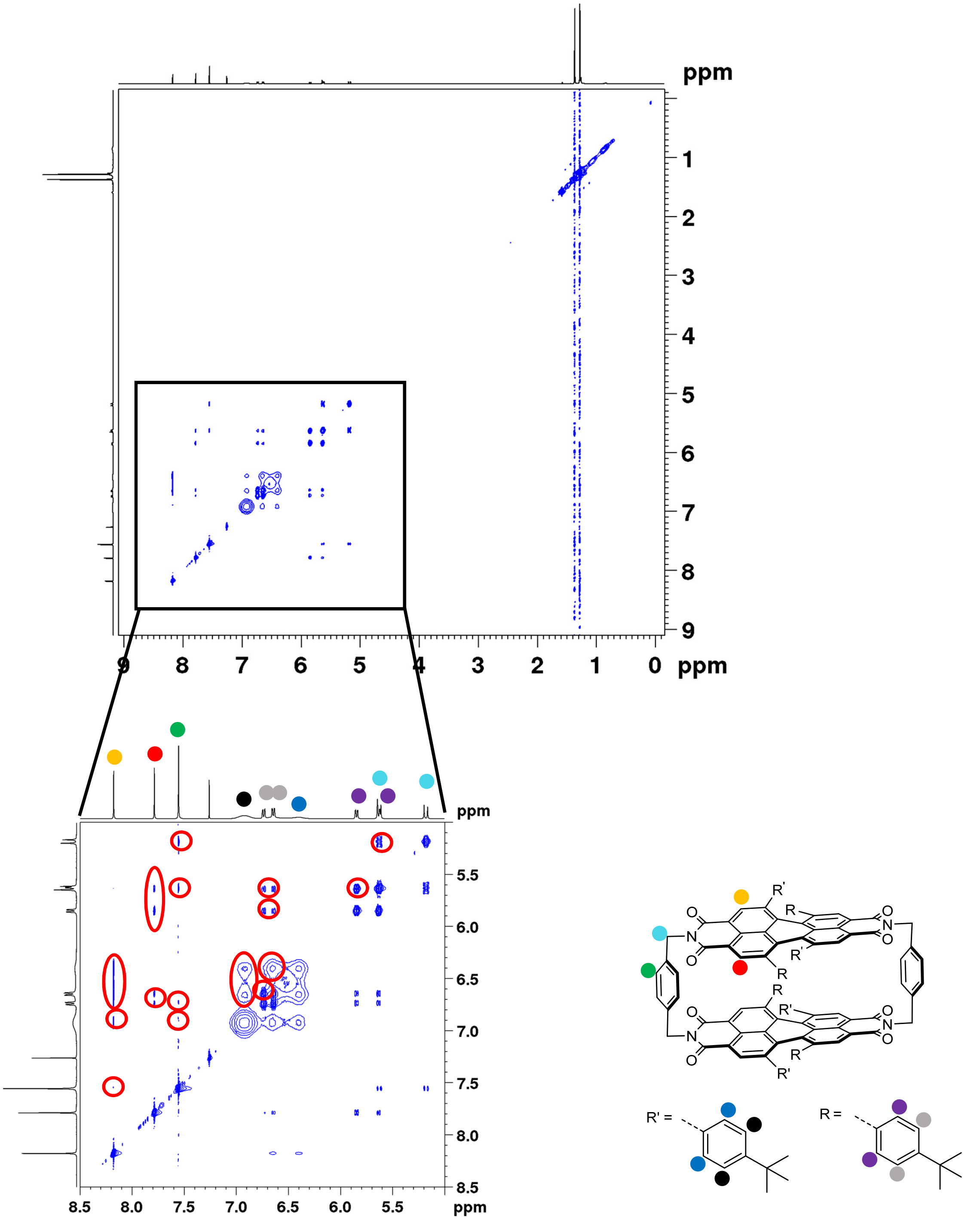

Supplementary Fig. 12 ^{1}H-^{1}H NOESY NMR spectrum (400 MHz, 295 K) of *rac*-**1** in $CDCl_3$. The important cross signals are marked in red.

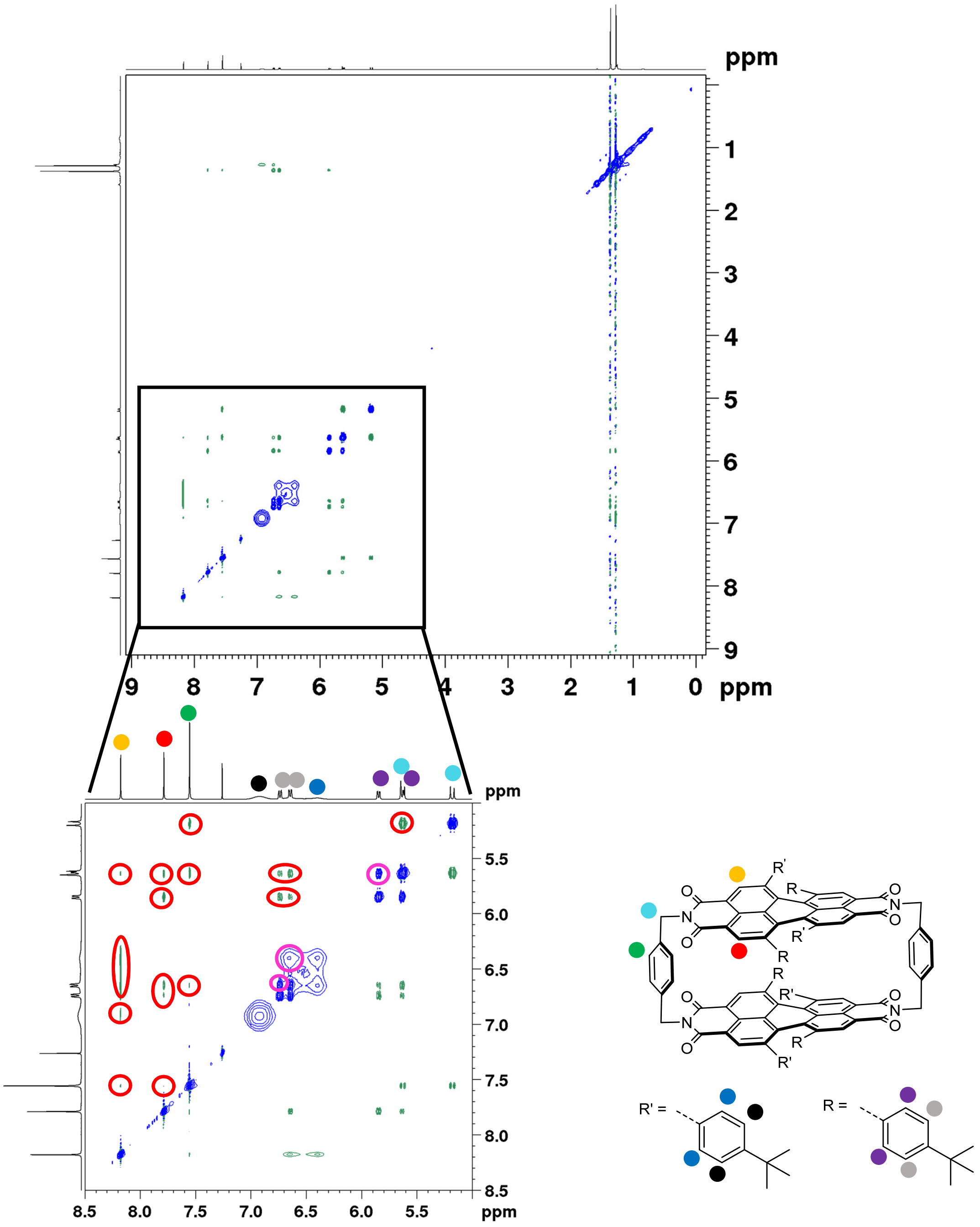

Supplementary Fig. 13 ^1H-^1H ROESY NMR spectrum (400 MHz, 295 K) of *rac*-**1** in $CDCl_3$. The important cross signals are marked in red. The exchange signals are marked in pink.

Supplementary Note 2

Compound *rac*-**1** shows the expected singlet for the aromatic *para*-xylylene spacer protons (green), whose close proximity to the diastereotopic methylene protons (turquoise) can be seen

from cross signals in the ^{1}H-^{1}H NOESY and ROESY spectra. The *ortho* protons of the PBI chromophore (red and orange) show two singlets in the proton NMR due to two different environments, i.e. one position that is more inside the cavity and one that points away from the cavity. The adjacent bay substituents can be easily assigned as they show through space correlations of different intensities to the *ortho* protons. Accordingly, the chemically and magnetically non-equivalent aromatic protons of the bay substituents (blue/black and purple/grey), which show spin-spin coupling signals in the ^{1}H-^{1}H COSY NMR, can be distinguished by different intensities of ^{1}H-^{1}H ROESY and NOESY cross signals (blue and purple are more intense than black and grey) with the protons marked in red and orange. In order to assign the *ortho* protons and their neighbouring bay substituents to the position that points outwards and towards the cyclophane cavity, we compared the shape of the aromatic bay substituents' proton signals. Both substituents show a rotation along the single bond to the PBI core with distinct exchange signals in the ^{1}H-^{1}H ROESY spectrum. While half of the substituents have broad signals, comparable to the monomeric reference dyes (Supplementary Fig. 2 and 4), the rest is characterized by sharp signals. Accordingly, we conclude that the "outer" substituents have a similar rotational barrier as in the case of the monomeric references, while the substituents that point towards the cavity are sharpened due to a more frozen rotation. Thus, the orange *ortho* proton must point away from the inner of the cyclophane while the red one is in close proximity to the cavity.

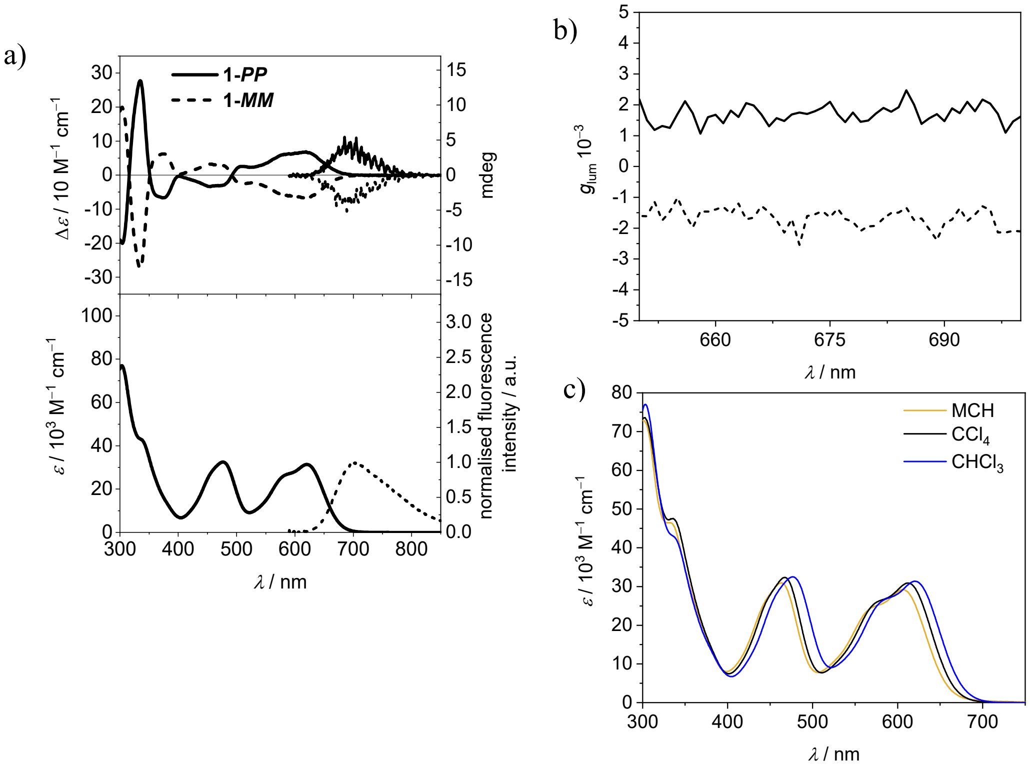

Supplementary Fig. 14 a) CD absorption spectra of **1-*PP*** and **1-*MM*** and UV/vis absorption spectrum of **1-*PP*** in chloroform at 295 K (c = 30 µM). Furthermore, the corresponding CPL (OD = 0.1) and fluorescence spectra (OD < 0.05) are shown. b) Plot of the dissymmetry factor g_{lum} of **1-*PP*** and **1-*MM*** in $CHCl_3$. c) UV/vis absorption spectra of *rac*-**1** in different solvents (c = 30 µM).

Supplementary Table 1. Fluorescence quantum yields of *rac*-**1** in different solvents, determined relative to Oxazin 1 ([7-(diethylamino)-phenoxazin-3-ylidene]-diethylazanium) in ethanol.

solvent	$CHCl_3$	CCl_4	MCH
**quantum yield** ϕ_f	0.23	0.41	0.38
k_r **[10⁷ s⁻¹]**	2.0	2.8	2.6
k_{nr} **[10⁷ s⁻¹]**	6.7	4.0	4.3

(rate constants determined according to $k_r = \phi/\tau$, $k_{nr} = 1/\tau - k_r$)

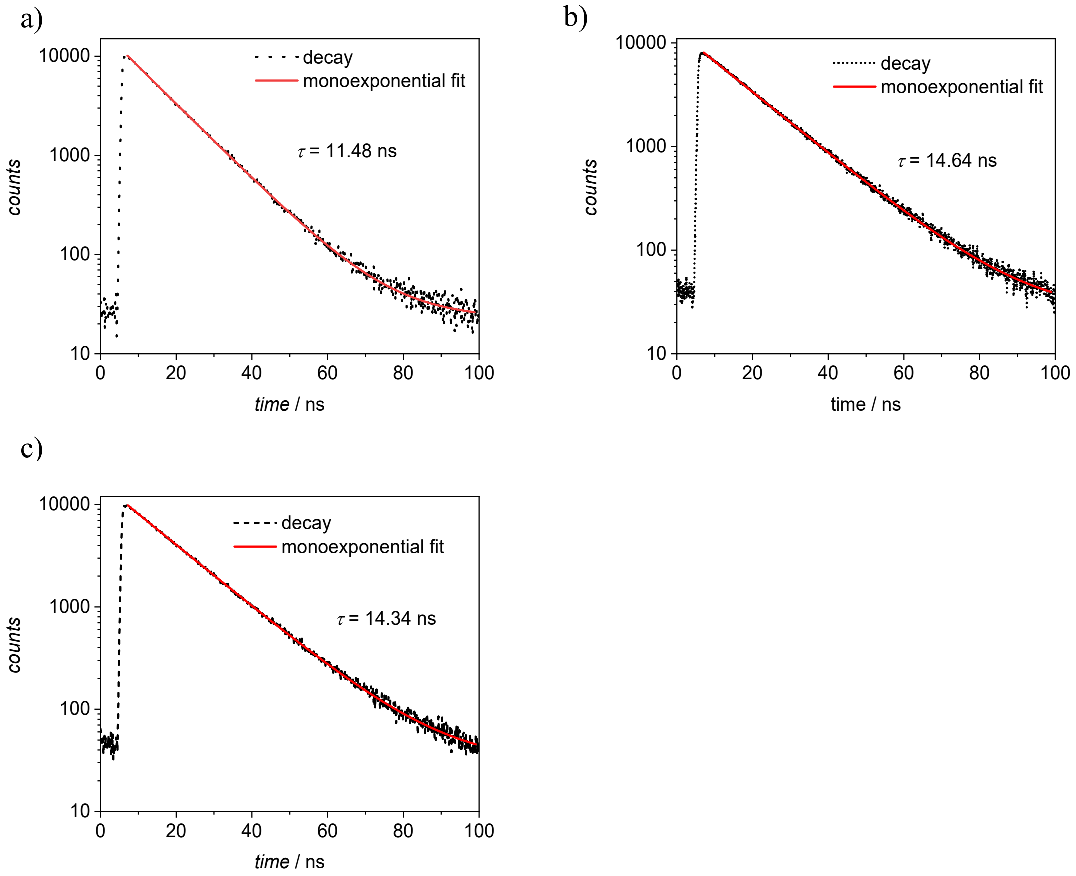

Supplementary Fig. 15. Lifetime measurement (black) of *rac*-**1** in a) $CHCl_3$ and b) tetrachloromethane and c) methylcyclohexane at 295 K (λ_{ex} = 635 nm, λ_{em} = 700 nm). The monoexponential fits are shown in red.

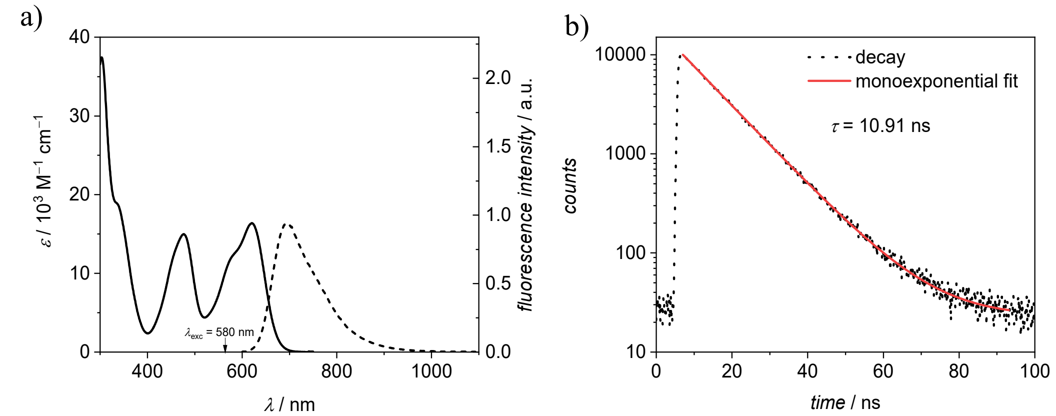

Supplementary Fig. 16 a) UV/vis absorption (c = 30 μM, solid line) and fluorescence (dashed line) spectra of *rac*-**3a** in chloroform and b) lifetime measurement (black) of *rac*-**3a** in $CHCl_3$ at 295 K (λ_{ex} = 635 nm, λ_{em} = 700 nm). The monoexponential fit is shown in red.

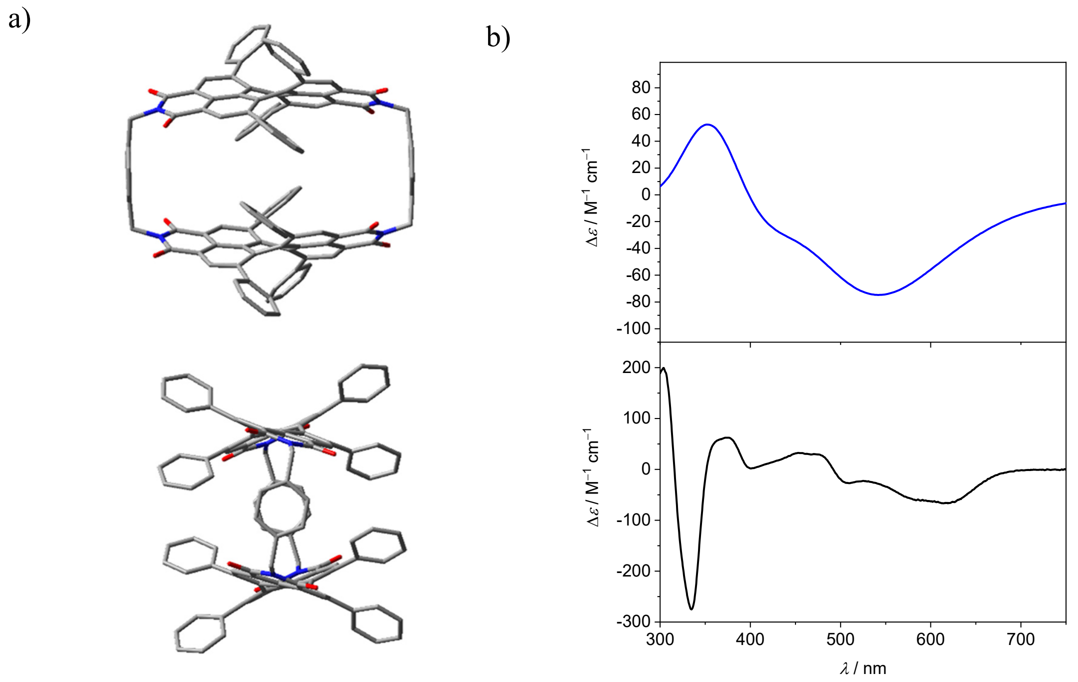

Supplementary Fig. 17 a) Energy minimized structures of **1-*MM*** (*tert*-butyl groups were removed for the calculation; hydrogen atoms are omitted for clarity). b) Calculated (blue solid line) and experimental (black solid line) CD spectra of the optimized structure and **1-*MM***.

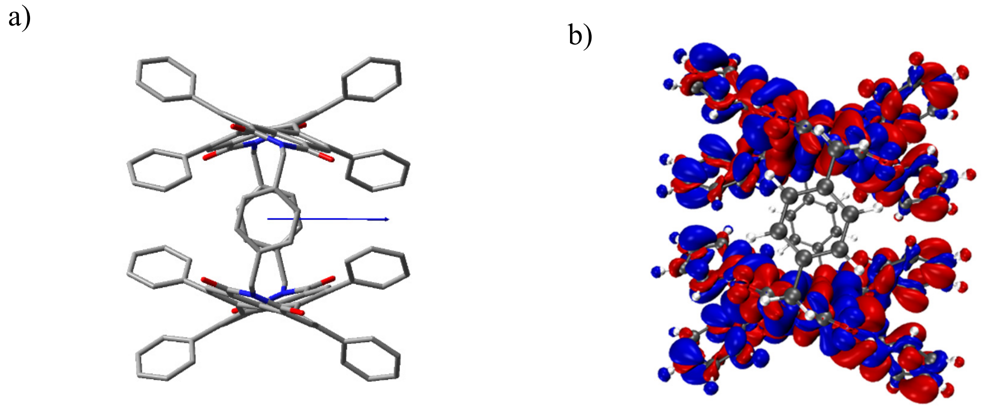

Supplementary Fig. 18 Energy minimized structure of **1-*MM*** (*tert*-butyl groups were removed for the calculation; hydrogen atoms are omitted for clarity) with a) transition dipole moment of the $S_0 \rightarrow S_2$ transition obtained by TD-DFT calculation (charge distribution vector × 0.5 from center of mass). b) Plotted transition densities for the $S_0 \rightarrow S_2$ transition.

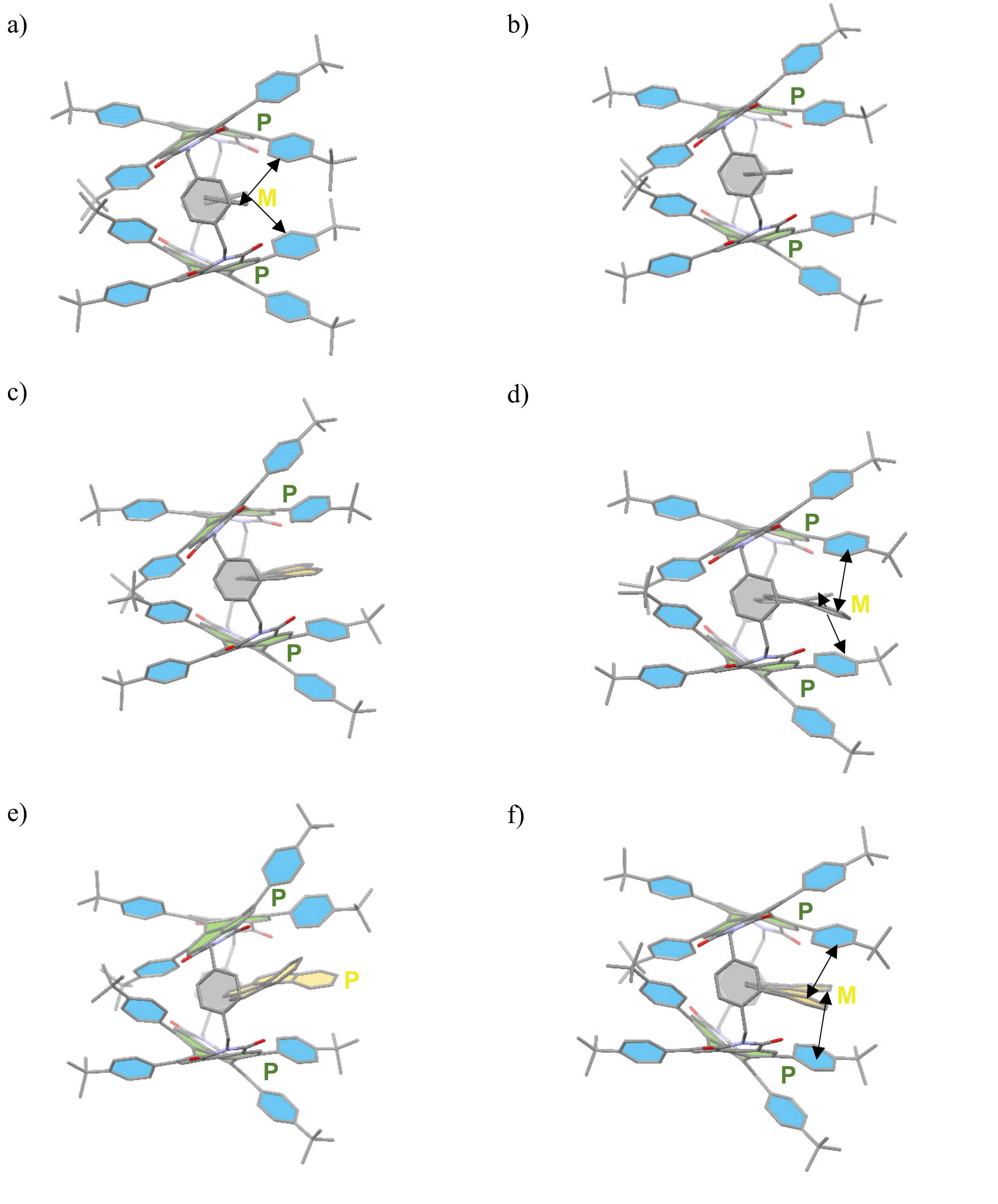

Supplementary Fig. 19 Front view of energy minimized complex structures of a) 1,1’-biphenyl⊂**1-*PP***, b) anthracene⊂**1-*PP***, c) pyrene⊂**1-*PP***, d) [4]helicene⊂**1-*PP*** e) *P*-[5]helicene⊂**1-*PP*** and f) perylene⊂**1-*PP*** (hydrogen atoms are omitted for clarity, the perylene units, the substituents, spacer units and the guests are highlighted in green, blue, grey and yellow).

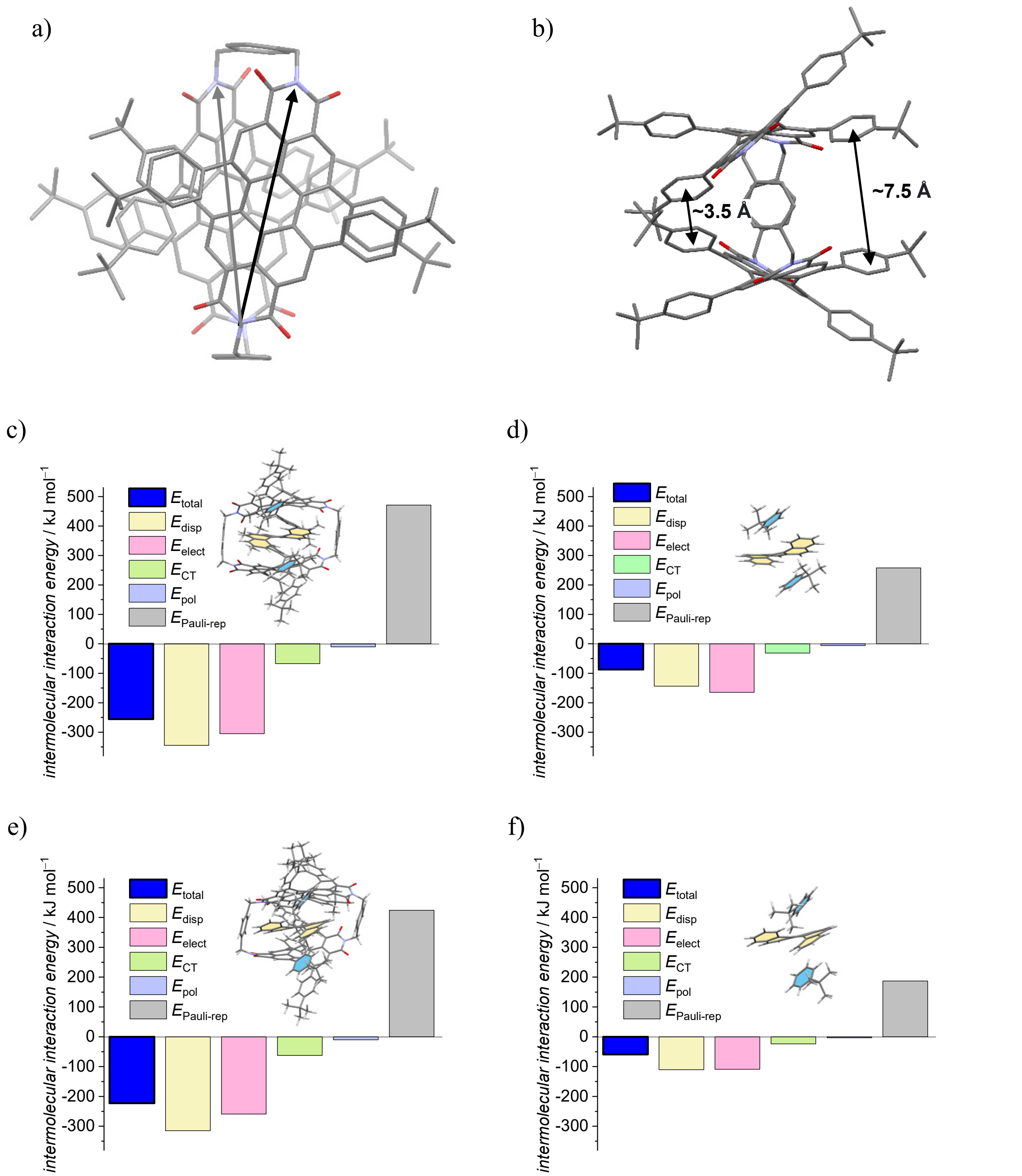

Supplementary Fig. 20 a) Top and b) front view of energy minimized complex structures of *M*-[5]helicene⊂**1-*PP***. The guest is omitted for clarity. c)-d) ALMO energy decomposition analysis performed on DFT optimized structure of *M*-[5]helicene⊂**1-*PP*** and a cutout of the complex structure (guest and the relevant bay substituent in close proximity as depicted in the inset). e)-f) ALMO energy decomposition analysis performed on DFT optimized structure of *P*-[5]helicene⊂**1-*PP*** and a cutout of the complex structure (guest and the relevant bay substituent in close proximity as depicted in the inset).

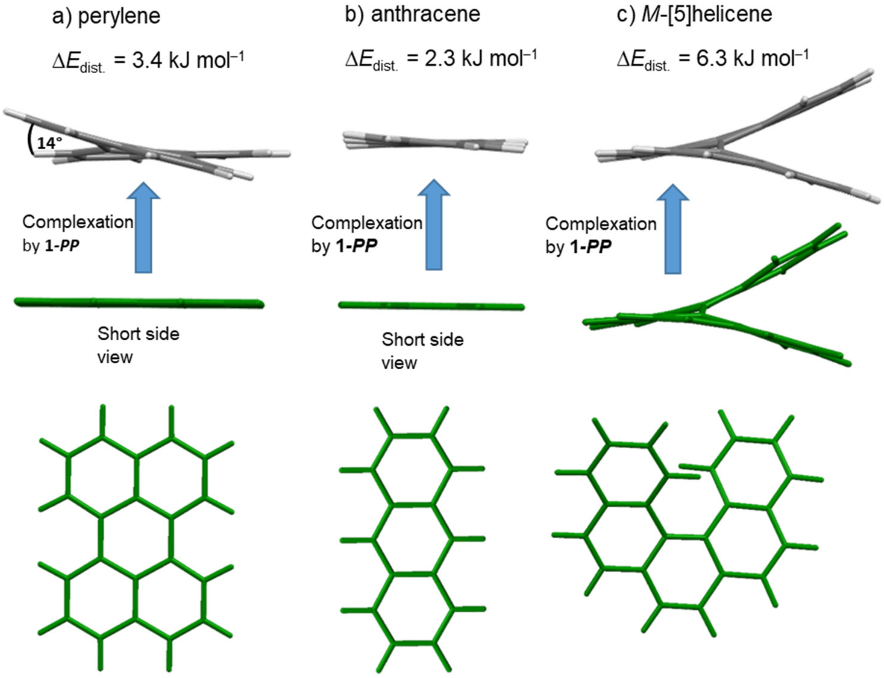

Supplementary Fig. 21 Geometric and energetic differences (SCF energies) between the free substrate (green) and the complexed substrate (grey) by **1-*PP***, given for a) perylene, b) anthracene and c) *M*-[5]helicene. The difference in SCF energies ($\Delta E_{dist.}$) shows that indeed only little energy is needed to distort perylene ($\Delta E_{dist.}$ = 3.4 kJ mol^{-1}) at the central benzene unit to significant dihedral angles of ~ 14° whilst for the other two examples similar or even higher energies are needed for more modest distortions.

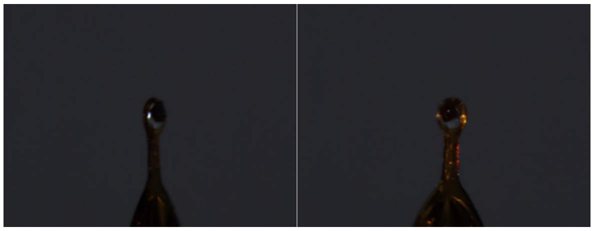

Supplementary Fig. 22 Images of the crystals of *rac*-**1**. The crystals of *rac*-**1** were grown from a chloroform by slow diffusion of methanol into the solution.

Supplementary Table 2. Crystal data and structure refinement for *rac*-**1**.

Identification code	CCDC 2207897
Empirical formula	$C_{145.76}H_{129.76}Cl_{15.29}N_4O_8$
Formula weight	2252.97
Temperature	100(2) K
Wavelength	1.54178 Å
Crystal system	monoclinic
Space group	C2/c
Unit cell dimensions	a = 31.824(2) Å
	b = 18.5816(12) Å
	c = 25.739(3) Å
Volume	12722.4(18) $Å^3$
Z	4
Density (calculated)	1.176 g/cm^3
Absorption coefficient	1.551 mm^{-1}
F(000)	4745
Crystal size	0.092 × 0.088 × 0.040 mm^3
Theta range for data collection	2.901 to 72.771°
Index ranges	$-39 \le h \le 39$, $-22 \le k \le 22$, $-31 \le l \le 31$
Reflections collected	94333
Independent reflections	12508 [R(int) = 0.1182]
Completeness to theta = 21.836°	99.6%
Absorption correction	Semi-empirical from equivalents
Refinement method	Full-matrix least-squares on F^2
Data / restraints / parameters	12508 / 19 / 789
Goodness-of-fit on F^2	1.137
Final R indices [$I > 2\sigma(I)$]	R_1 = 0.0893, wR_2 = 0.1911
R indices (all data)	R_1 = 0.1109, wR_2 = 0.2013

The crystals of 1,1'-biphenyl⊂*rac*-**1** were grown from a saturated and filtered host and guest solution in methylcyclohexane in a borosilicate glass tube (10 mm × 75 mm), which was put into a closed vial filled with methanol.

Supplementary Table 3. Crystal data and structure refinement for1,1'-biphenyl⊂*rac*-**1**.

Identification code	CCDC 2207898
Empirical formula	$C_{198.73} H_{175.02} N_4 O_8$
Formula weight	2747.23
Temperature	100(2) K
Wavelength	0.61992 Å
Crystal system	Triclinic
Space group	$P\bar{1}$
Unit cell dimensions	a = 20.835(4) Å α = 87.655(3)°
	b = 24.770(5) Å β = 79.275(8)°
	c = 31.712(5) Å γ = 72.772(13)°
Volume	15357(5) Å^3
Z	4
Density (calculated)	1.188 g/cm^3
Absorption coefficient	0.055 mm^{-1}
F(000)	5837.6
Crystal size	0.100 × 0.100 × 0.100 mm^3
Theta range for data collection	0.570 to 27.993°.
Index ranges	$-27 \le h \le 27$, $-37 \le k \le 37$, $-48 \le l \le 47$
Reflections collected	590152
Independent reflections	85764 [R(int) = 0.0920]
Completeness to theta = 21.836°	99.0 %
Absorption correction	None
Refinement method	Full-matrix least-squares on F^2
Data / restraints / parameters	85764 / 4753 / 4684
Goodness-of-fit on F^2	1.028
Final R indices [$I > 2\sigma(I)$]	R_1 = 0.0996, wR_2 = 0.2656
R indices (all data)	R_1 = 0.1615, wR_2 = 0.3232
Extinction coefficient	n/a
Largest diff. peak and hole	0.658 and –0.328 e·Å^{-3}

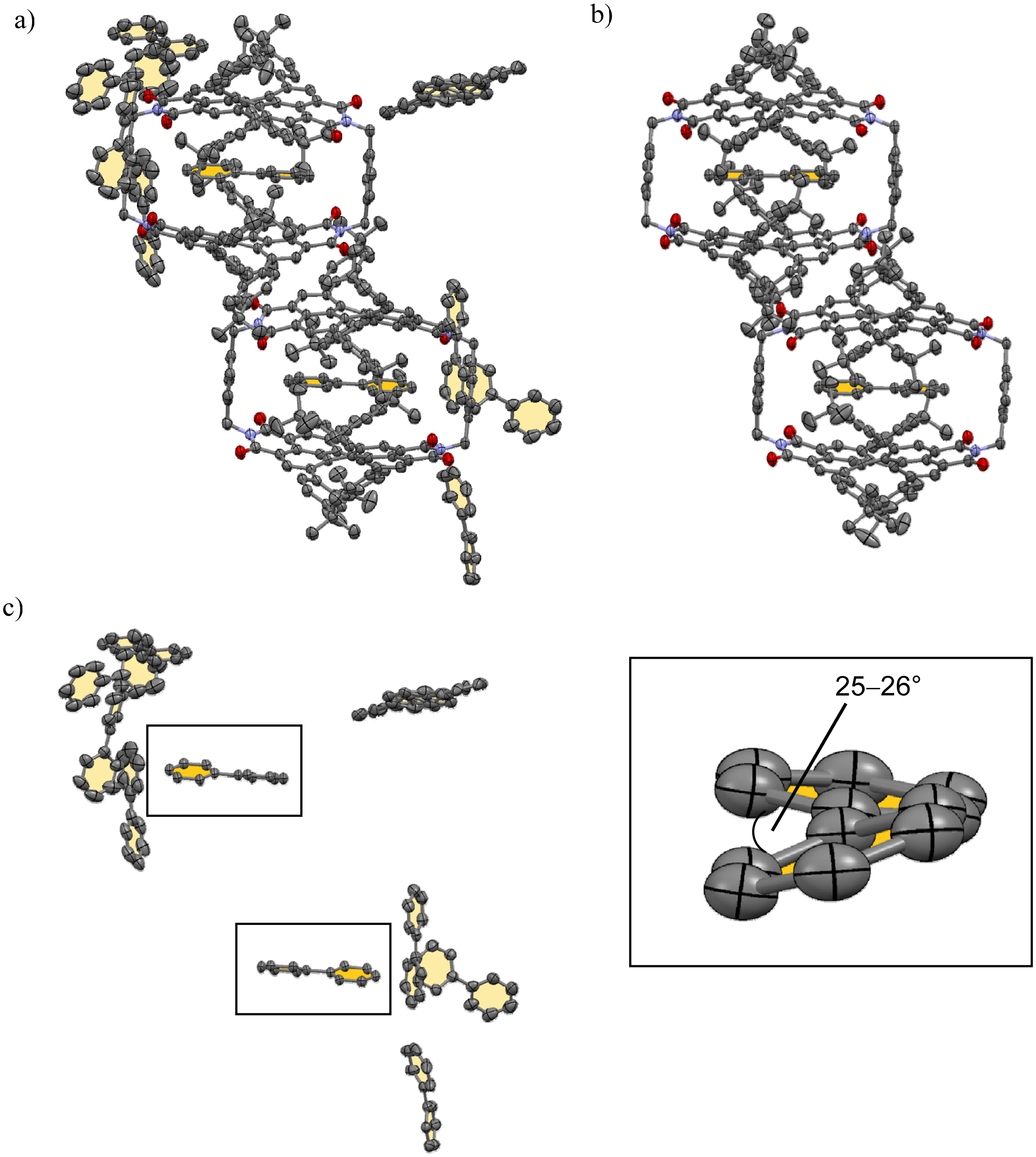

Supplementary Fig. 23 Side view of the packing of 1,1'-biphenyl⊂ *rac*-**1** in the solid state, obtained by single crystal X-ray analysis a) with and b) without the surrounding 1,1'-biphenyl matrix. c) Solid state structure of 1,1'-biphenyl⊂ *rac*-**1** without host molecules and zoom-in of the complexed 1,1'-biphenyl guests within the host cavities.

Supplementary Note 3

From the solid state structure of the host-guest complex, the importance of guest flexibility investigated in the first part of this article also becomes apparent. While the rigidity of the PBI units is manifested in an unchanged core twist of 34–37° as in the solid state structure of the

free host (Fig. 2), we can clearly see how the angle, that the two planes of the phenyl units of the 1,1'-biphenyl guest spans, adapt to the host cavity. Thus, the twist of the phenyl moieties of the host-embedded 1,1'-biphenyl amounts to an angle of 25–26° (Supplementary Fig. 23c), which is in good accordance with the result from our DFT calculation (23°, Supplementary Fig. 19a), while most of the surrounding biphenyl molecules in this crystal show either a twist angle of around 40° or planarity.

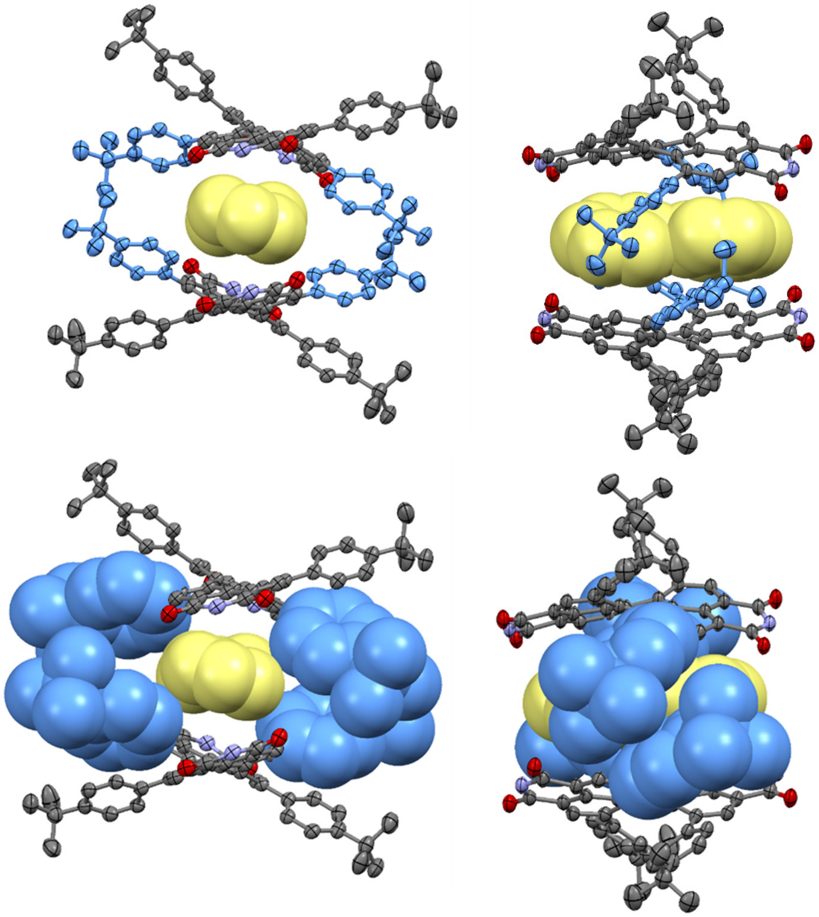

Supplementary Fig. 24 Front and side view of 1,1'-biphenyl⊂ **1-*PP*** in the solid state, obtained by single crystal X-ray analysis and a depiction of the structure with the relevant bay substituents and the guest in the space filling model in order to visualize the origin of the heterochiral guest recognition. The guest molecule and the relevant bay substituents are highlighted in yellow and blue respectively. The *para*-xylylene spacer and hydrogens are omitted for clarity.

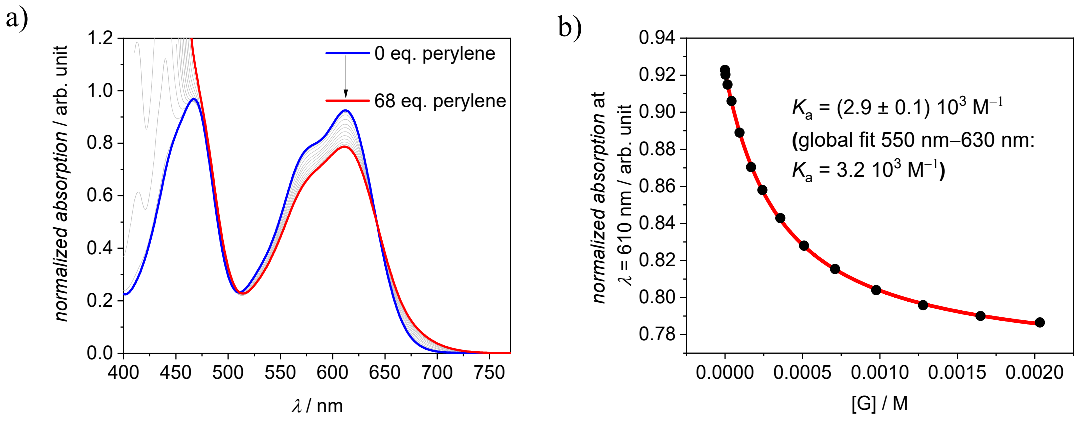

Supplementary Fig. 25 a) UV/vis spectra of cyclophane *rac*-**1** in CCl_4 at 22 °C ($c = 30 \times 10^{-6}$ M) upon the addition of perylene as a guest and b) the resulting plot of the absorption at $\lambda = 610$ nm with nonlinear curve fit (1:1 binding model, red curve) and binding constants obtained from this curve fit as well as a global fit for the whole $S_0 \rightarrow S_1$ absorption band of the PBI.

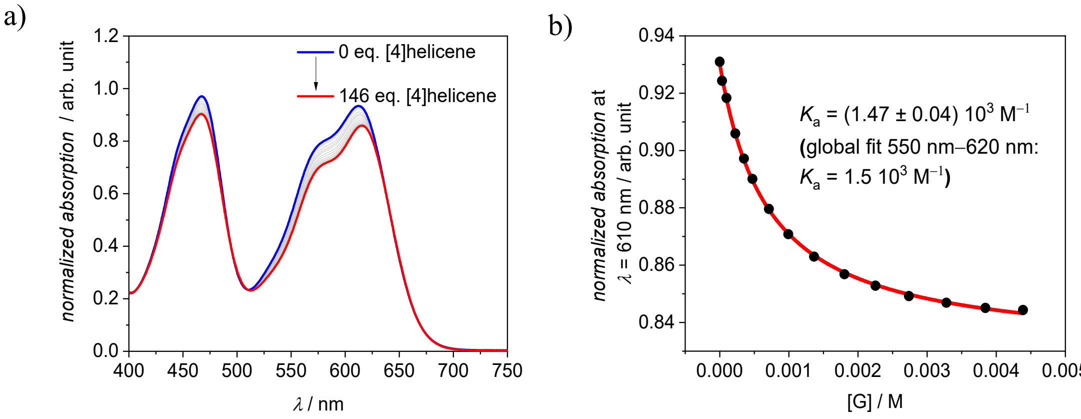

Supplementary Fig. 26 a) UV/vis spectra of cyclophane *rac*-**1** in CCl_4 at 22 °C ($c = 30 \times 10^{-6}$ M) upon the addition of [4]helicene as a guest and b) the resulting plot of the absorption at $\lambda = 610$ nm with nonlinear curve fit (1:1 binding model, red curve) and binding constants obtained from this curve fit as well as a global fit for the whole $S_0 \rightarrow S_1$ absorption band of the PBI.

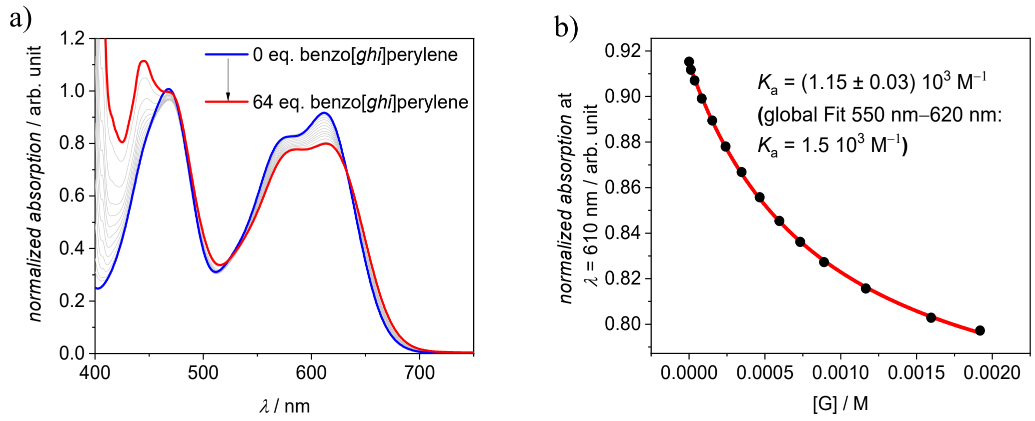

Supplementary Fig. 27 a) UV/vis spectra of cyclophane *rac*-**1** in CCl_4 at 22 °C ($c = 30 \times 10^{-6}$ M) upon the addition of benzo[*ghi*]perylene as a guest and b) the resulting plot of the absorption at $\lambda = 610$ nm with nonlinear curve fit (1:1 binding model, red curve) and binding constants obtained from this curve fit as well as a global fit for the whole $S_0 \rightarrow S_1$ absorption band of the PBI.

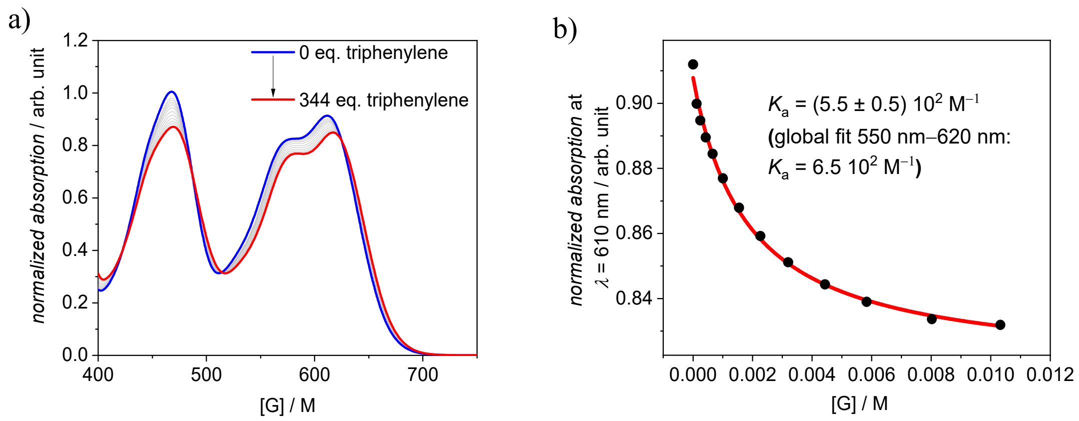

Supplementary Fig. 28 a) UV/vis spectra of cyclophane *rac*-**1** in CCl_4 at 22 °C ($c = 30 \times 10^{-6}$ M) upon the addition of triphenylene as a guest and b) the resulting plot of the absorption at $\lambda = 610$ nm with nonlinear curve fit (1:1 binding model, red curve) and binding constants obtained from this curve fit as well as a global fit for the whole $S_0 \rightarrow S_1$ absorption band of the PBI.

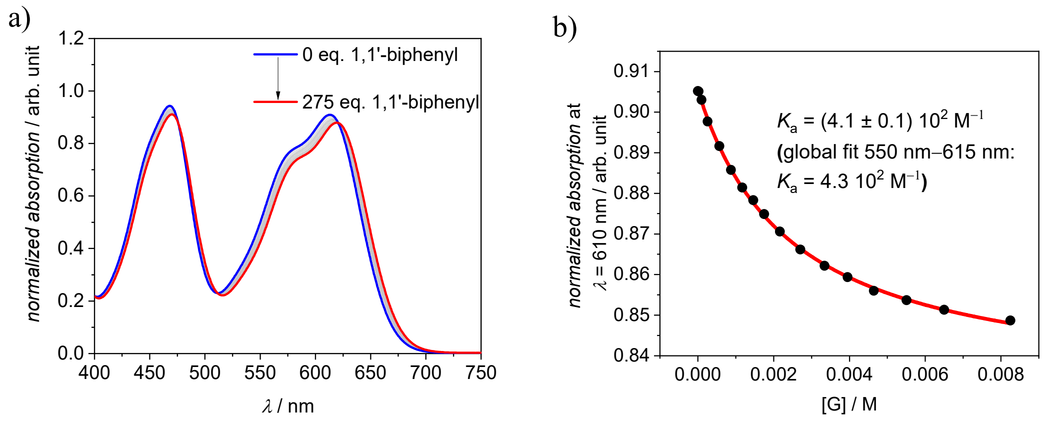

Supplementary Fig. 29 a) UV/vis spectra of cyclophane *rac*-**1** in CCl_4 at 22 °C ($c = 30 \times 10^{-6}$ M) upon the addition of 1,1'-biphenyl as a guest and b) the resulting plot of the absorption at $\lambda = 610$ nm with nonlinear curve fit (1:1 binding model, red curve) and binding constants obtained from this curve fit as well as a global fit for the whole $S_0 \rightarrow S_1$ absorption band of the PBI.

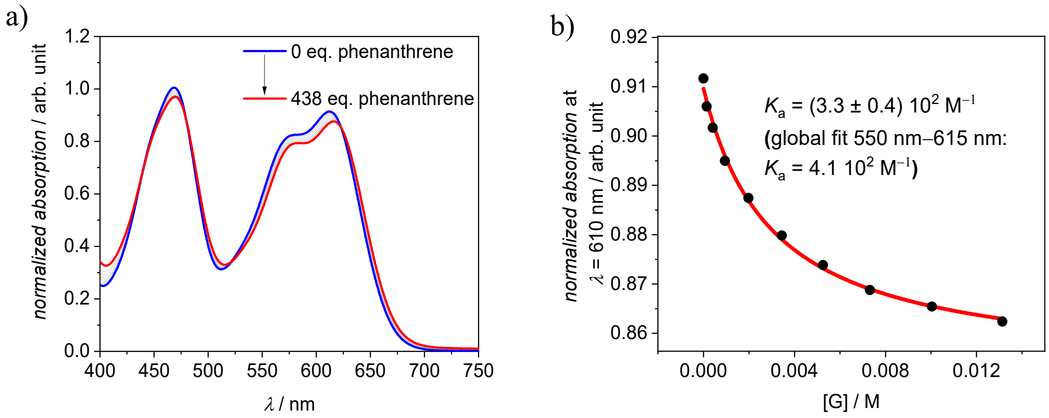

Supplementary Fig. 30 a) UV/vis spectra of cyclophane *rac*-**1** in CCl_4 at 22 °C ($c = 30 \times 10^{-6}$ M) upon the addition of phenanthrene as a guest and b) the resulting plot of the absorption at $\lambda = 610$ nm with nonlinear curve fit (1:1 binding model, red curve) and binding constants obtained from this curve fit as well as a global fit for the whole $S_0 \rightarrow S_1$ absorption band of the PBI.

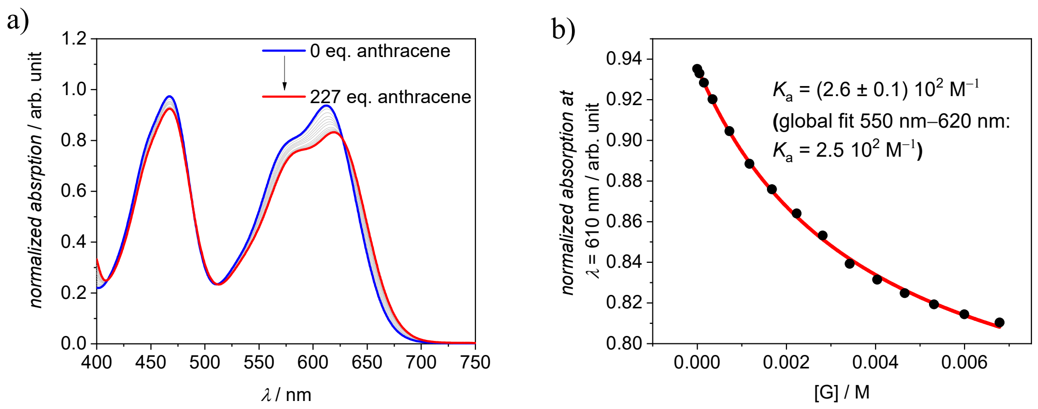

Supplementary Fig. 31 a) UV/vis spectra of cyclophane *rac*-**1** in CCl_4 at 22 °C ($c = 30 \times 10^{-6}$ M) upon the addition of anthracene as a guest and b) the resulting plot of the absorption at $\lambda = 610$ nm with nonlinear curve fit (1:1 binding model, red curve) and binding constants obtained from this curve fit as well as a global fit for the whole $S_0 \rightarrow S_1$ absorption band of the PBI.

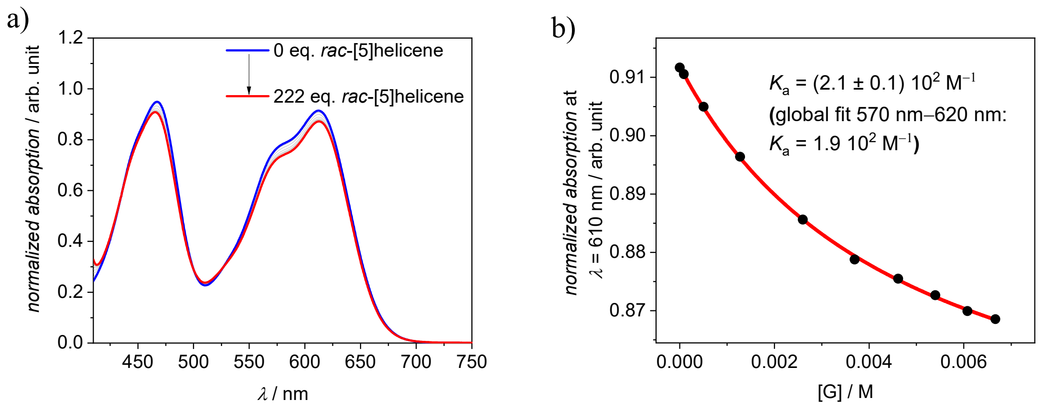

Supplementary Fig. 32 a) UV/vis spectra of cyclophane *rac*-**1** in CCl_4 at 22 °C ($c = 30 \times 10^{-6}$ M) upon the addition of [5]helicene as a guest and b) the resulting plot of the absorption at $\lambda = 610$ nm with nonlinear curve fit (1:1 binding model, red curve) and binding constants obtained from this curve fit as well as a global fit for the whole $S_0 \rightarrow S_1$ absorption band of the PBI.

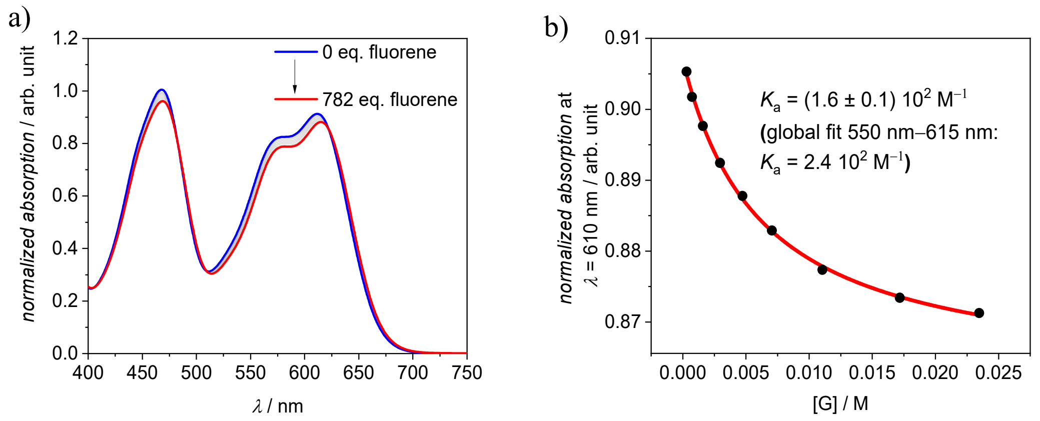

Supplementary Fig. 33 a) UV/vis spectra of cyclophane *rac*-**1** in CCl_4 at 22 °C ($c = 30 \times 10^{-6}$ M) upon the addition of fluorene as a guest and b) the resulting plot of the absorption at $\lambda = 610$ nm with nonlinear curve fit (1:1 binding model, red curve) and binding constants obtained from this curve fit as well as a global fit for the whole $S_0 \rightarrow S_1$ absorption band of the PBI.

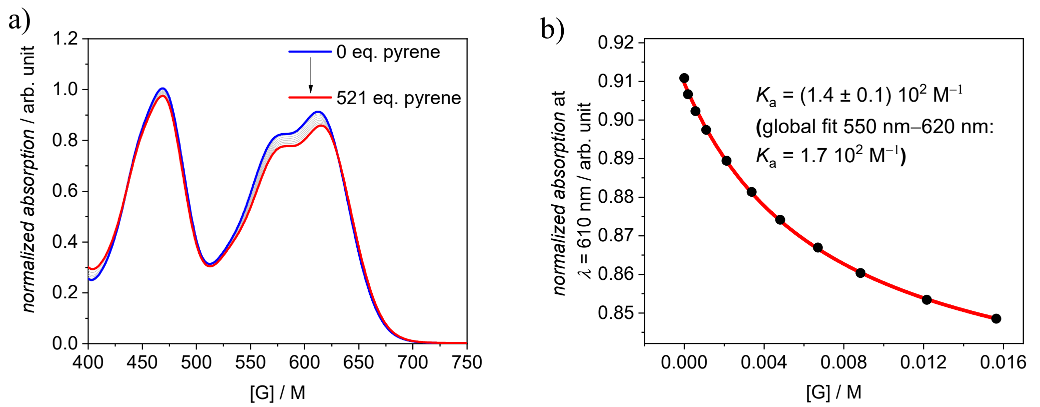

Supplementary Fig. 34 a) UV/vis spectra of cyclophane *rac*-**1** in CCl_4 at 22 °C ($c = 30 \times 10^{-6}$ M) upon the addition of pyrene as a guest and b) the resulting plot of the absorption at $\lambda = 610$ nm with nonlinear curve fit (1:1 binding model, red curve) and binding constants obtained from this curve fit as well as a global fit for the whole $S_0 \rightarrow S_1$ absorption band of the PBI.

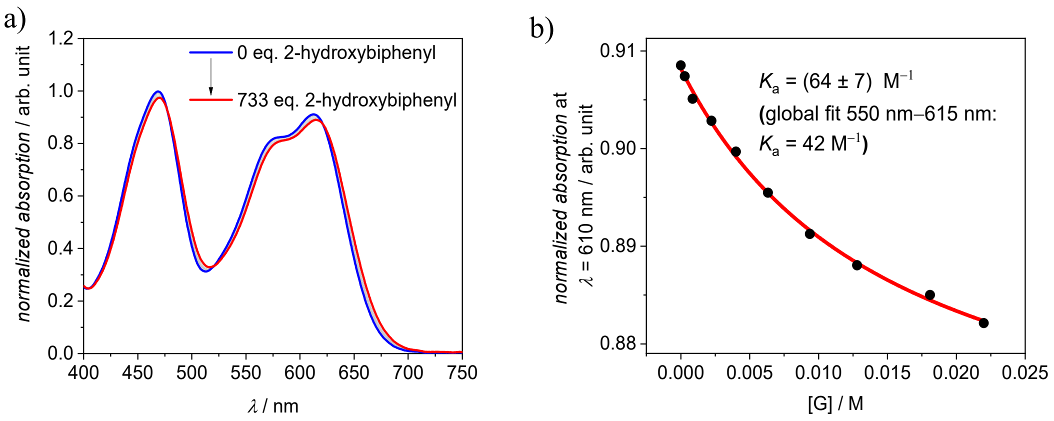

Supplementary Fig. 35 a) UV/vis spectra of cyclophane *rac*-**1** in CCl_4 at 22 °C ($c = 30 \times 10^{-6}$ M) upon the addition of 2-hydroxybiphenyl as a guest and b) the resulting plot of the absorption at $\lambda = 610$ nm with nonlinear curve fit (1:1 binding model, red curve) and binding constants obtained from this curve fit as well as a global fit for the whole $S_0 \rightarrow S_1$ absorption band of the PBI.

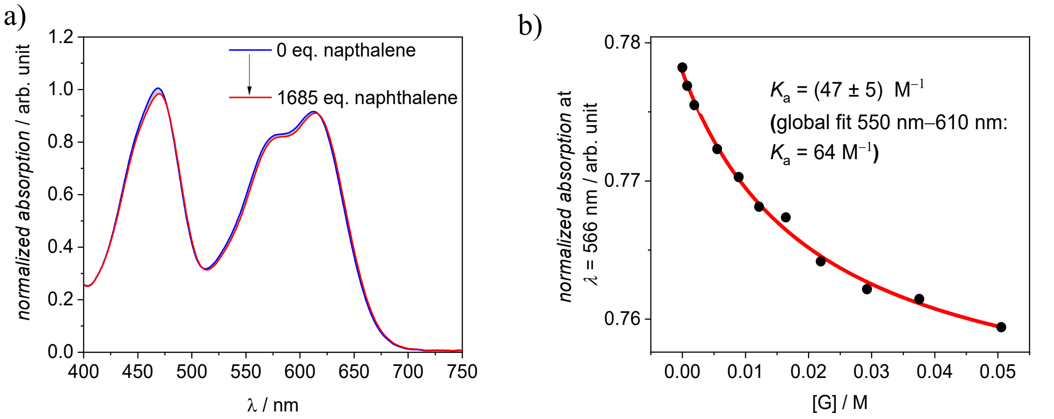

Supplementary Fig. 36 a) UV/vis spectra of cyclophane *rac*-**1** in CCl_4 at 22 °C ($c = 30 \times 10^{-6}$ M) upon the addition of naphthalene as a guest and b) the resulting plot of the absorption at $\lambda = 566$ nm with nonlinear curve fit (1:1 binding model, red curve) and binding constants obtained from this curve fit as well as a global fit for the whole $S_0 \rightarrow S_1$ absorption band of the PBI.

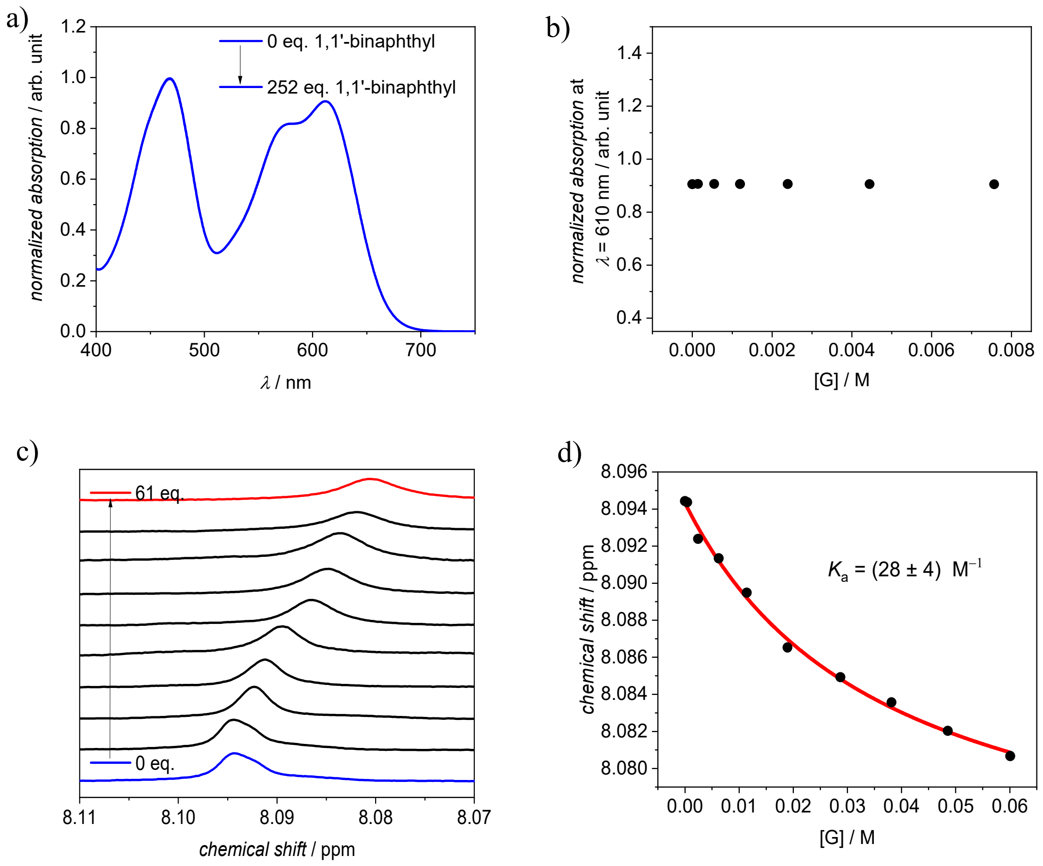

Supplementary Fig. 37 a) UV/vis spectra of cyclophane *rac*-**1** in CCl_4 at 22 °C ($c = 30 \times 10^{-6}$ M) upon the addition of 1,1'-binaphthyl as a guest and b) the resulting plot of the absorption at $\lambda = 566$ nm. c) Excerpt of ^{1}H NMR (CCl_4/MCH-d_{14} 3:1 (v:v), c (*rac*-**1**) = 1.0×10^{-3} M, 295 K) titration experiment of host *rac*-**1** with 1,1'-binaphthyl and d) the nonlinear curve fit of the obtained chemical shift versus guest concentration according to the 1:1 binding model.

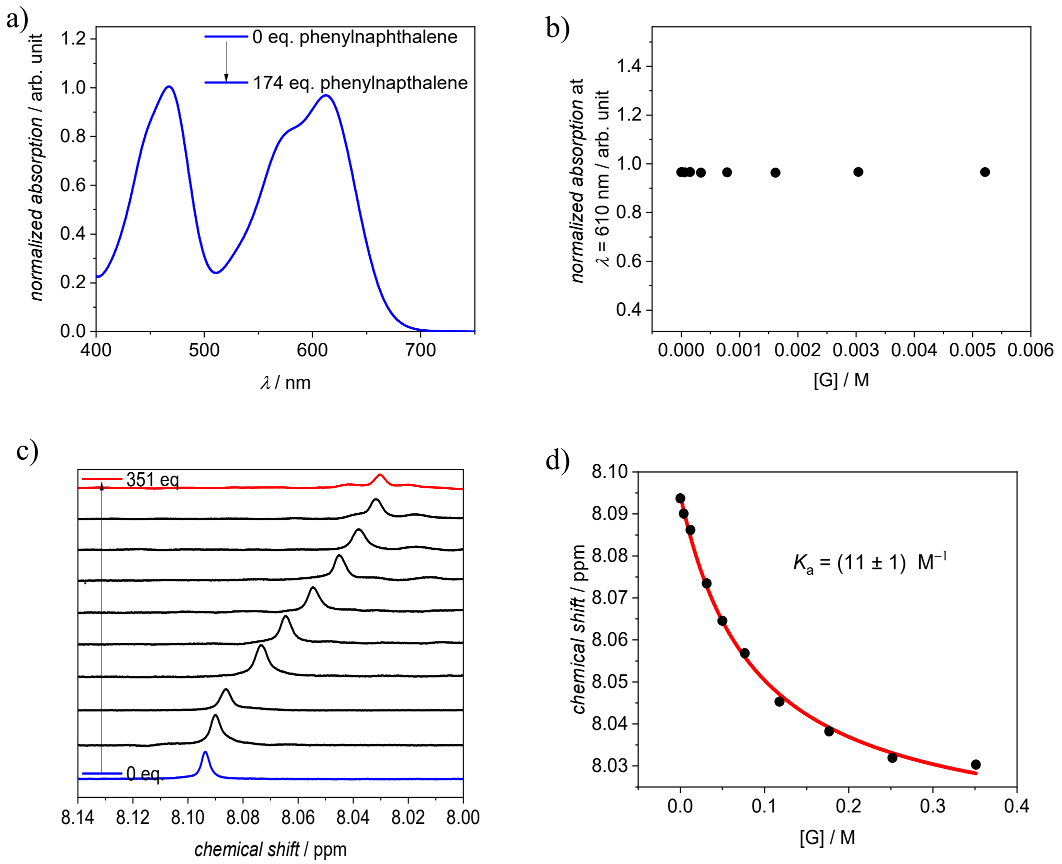

Supplementary Fig. 38 a) UV/vis spectra of cyclophane *rac*-**1** in CCl_4 at 22 °C ($c = 30 \times 10^{-6}$ M) upon the addition of phenylnaphthalene as a guest and b) the resulting plot of the absorption at $\lambda = 566$ nm. c) Excerpt of ^{1}H NMR (CCl_4/MCH-d_{14} 3:1 (v:v), c (*rac*-**1**) = 1.0×10^{-3} M, 295 K) titration experiment of host *rac*-**1** with phenylnapthalene and d) the nonlinear curve fit of the obtained chemical shift versus guest concentration according to the 1:1 binding model.

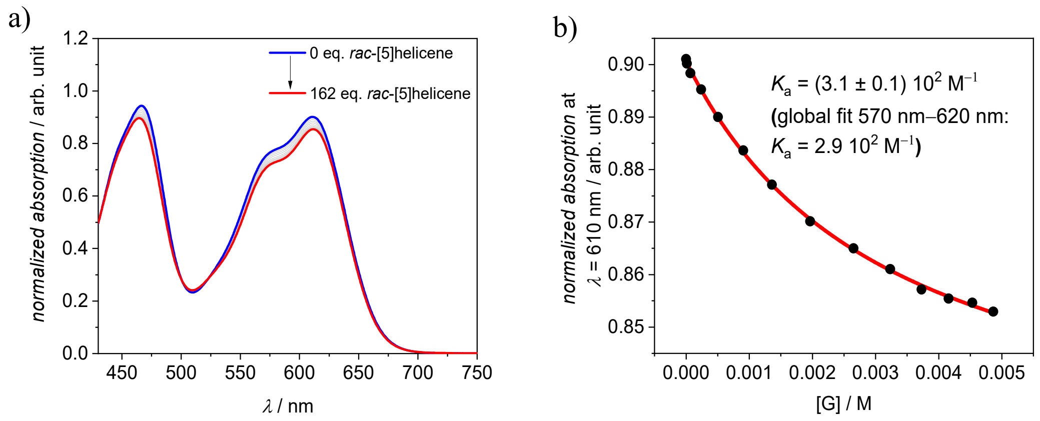

Supplementary Fig. 39 a) UV/vis spectra of cyclophane *rac*-**1** in CCl_4/MCH 3:1 (v:v) at 22 °C ($c = 30 \times 10^{-6}$ M) upon the addition of *rac*-[5]helicene as a guest and b) the resulting plot of the absorption at λ = 610 nm with nonlinear curve fit (1:1 binding model, red curve) and binding constants obtained from this curve fit as well as a global fit for the whole $S_0 \rightarrow S_1$ absorption band of the PBI.

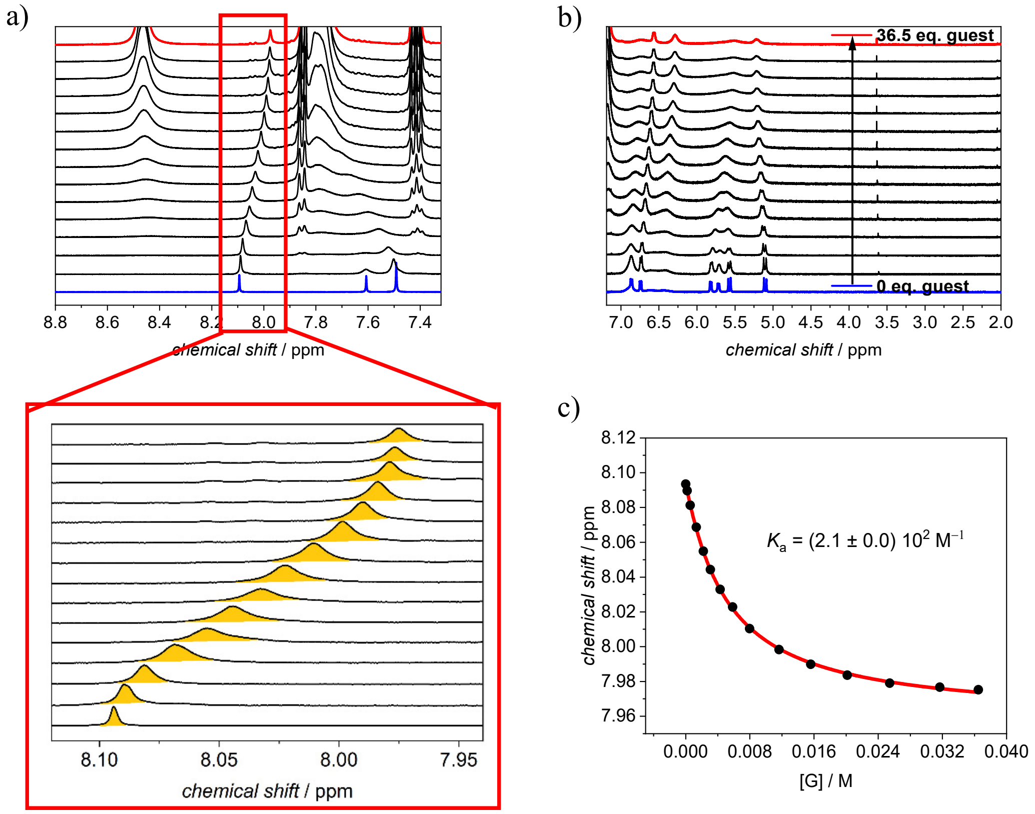

Supplementary Fig. 40 a)-b) Excerpts of ^{1}H NMR (CCl_4/MCH-d_{14} 3:1 (v:v), c (*rac*-**1**) = 1.0×10^{-3} M, 295 K) titration experiment of host *rac*-**1** with *rac*-[5]helicene. A zoom-in is shown in order to visualize the upfield shift of the perylene proton upon guest addition, which was used for c) the nonlinear curve fit of the obtained chemical shift versus guest concentration according to the 1:1 binding model.

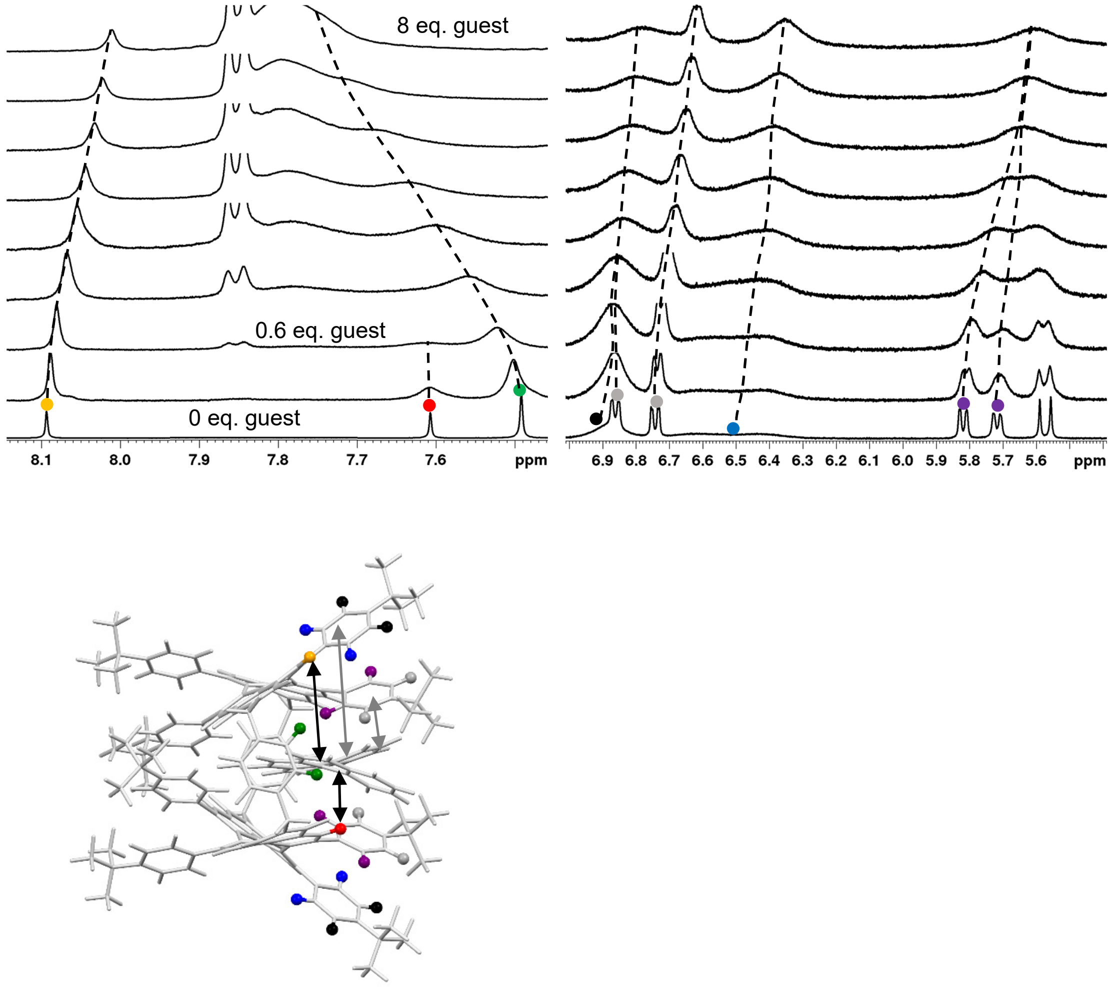

Supplementary Fig. 41 Excerpts of ^{1}H NMR (CCl_4/MCH-d_{14} 3:1 (v:v), *c* (*rac*-**1**) = 1.0 × 10^{-3} M, 295 K) titration experiment from Supplementary Fig. 40 of host *rac*-**1** with *rac*-[5]helicene and DFT optimized structure of *M*-[5]helicene⊂**1-*PP***. The black and grey double arrows indicate the different distance between the guest and the aromatic protons of the phenyl substituents and the perylene *ortho* protons.

Supplementary Note 4

First, we see a continuous downfield shift of the aromatic xylylene bridge protons upon addition of [5]helicene as these are oriented perpendicular to the aromatic surface of the guest and hence shifted to higher chemical shifts as a result of the guest's ring current. Second, the two non-equivalent perylene *ortho* protons are at very different distances to the guest molecule. As a result, the more distant proton (orange) is only weakly upfield-shifted due to the shielding of the guest's ring current, while the closer proton (red) is extremely broadened already after the addition of 0.6 eq. of guest. Similar, but less pronounced observations, can be made for the bay substituents where the outer phenyl units experience almost no shielding or deshielding

upon guest complexation (protons marked in blue and black). In contrast, we see more distinct shifts and signal broadening of the substituents which are closer to the cavity (protons marked in purple and grey).

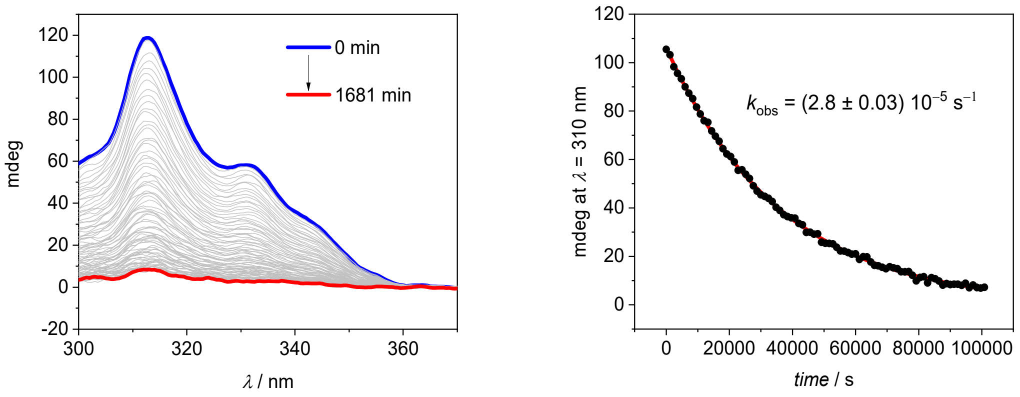

Supplementary Fig. 42 Time-dependent CD spectrum of *P*-[5]helicene (c = 300 μM) in CCl_4/MCH-d_{14} 3:1 at 295 K.

$$S_t = \Delta S e^{-k_{obs}t} + S_\infty \quad \text{(S2)}$$

(S: chemical shift or CD signal)[9,10]

$$\Delta G^{\ddagger} = -RT\ln\left(\frac{k_e h}{k_b T}\right) \quad \text{(S3)}$$

(with $k_{obs} = k_{rac} = 2k_e$ (enantiomerization rate constant), R: ideal gas constant, h: Planck constant, k_b: Boltzmann constant, $T = 295$ K)[11]

Accordingly, we obtain for $\Delta G^{\ddagger}$= 99.6 kJ/mol with $k_e = 1.4 \times 10^{-5}$ s^{-1} (in the absence of the host) and $\Delta G^{\ddagger}$= 99.0 kJ/mol with $k_e = 1.8 \times 10^{-5}$ s^{-1} (in the presence of the host).

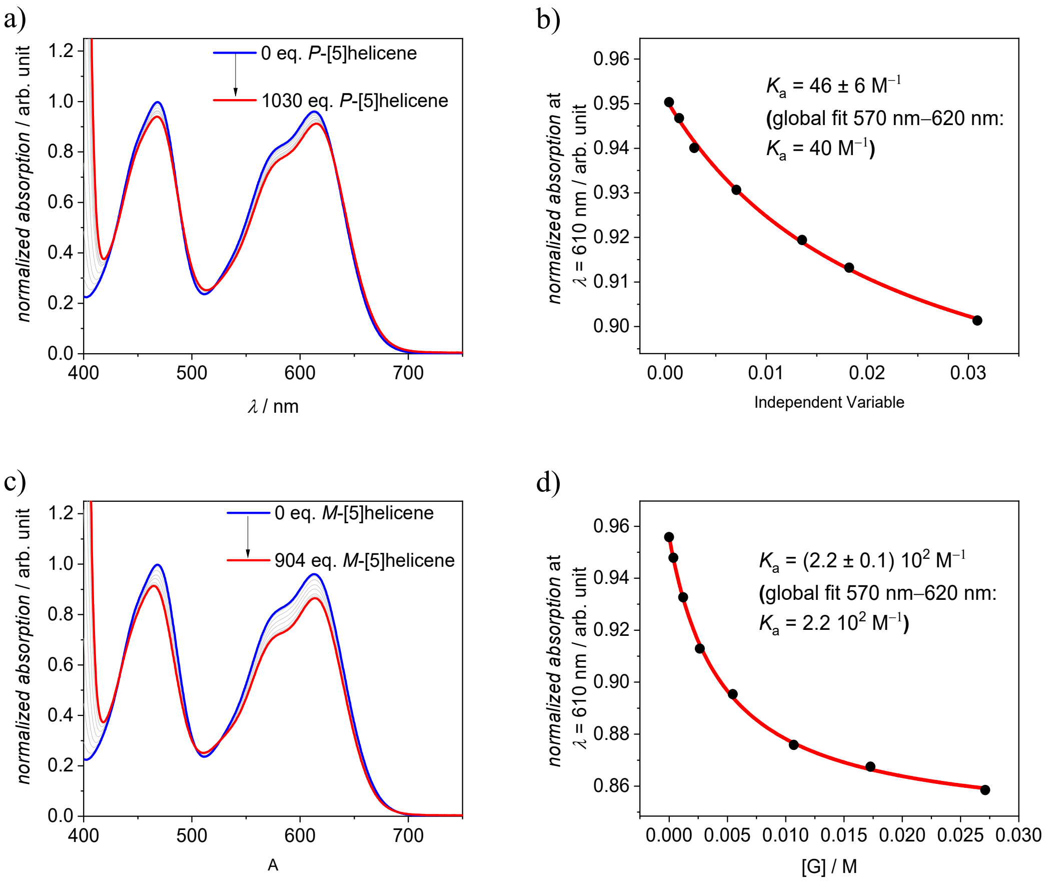

Supplementary Fig. 43 a) UV/vis spectra of cyclophane **1-*PP*** in CCl_4 at 22 °C ($c = 30 \times 10^{-6}$ M) upon the addition of *P*-[5]helicene as a guest and b) the resulting plot of the absorption at $\lambda = 610$ nm with nonlinear curve fit (1:1 binding model, red curve) and binding constants obtained from this curve fit as well as a global fit for the whole $S_0 \rightarrow S_1$ absorption band of the PBI. c) UV/vis spectra of cyclophane **1-*PP*** in CCl_4 at 22 °C ($c = 30 \times 10^{-6}$ M) upon the addition of *M*-[5]helicene as a guest and d) the resulting plot of the absorption at $\lambda = 610$ nm with nonlinear curve fit (1:1 binding model, red curve) and binding constants obtained from this curve fit as well as a global fit for the whole $S_0 \rightarrow S_1$ absorption band of the PBI.

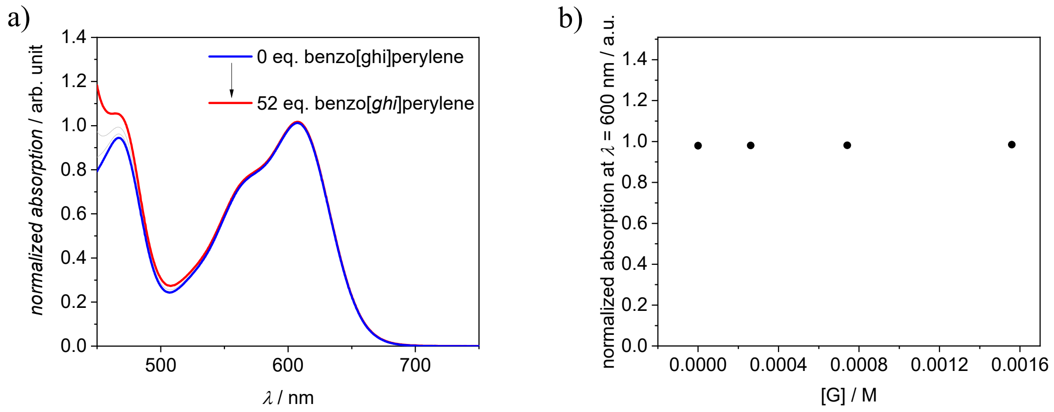

Supplementary Fig. 44 a) UV/vis spectra of *rac*-**3a** in CCl_4 at 22 °C (c = 30 × 10^{-6} M) upon the addition of benzo[*ghi*]perylene and b) the resulting plot of the absorption at λ = 600 nm.

Supplementary References

1. Renner, R., Mahlmeister, B., Anhalt, O., Stolte, M. & Würthner, F. Chiral Perylene Bisimide Dyes by Interlocked Arene Substituents in the Bay Area. *Chem. Eur. J.* **27**, 11997–12006, (2021).
2. Dubey, R. K., Westerveld, N., Grozema, F. C., Sudhölter, E. J., & Jager, W. F. Facile synthesis of pure 1, 6, 7, 12-tetrachloroperylene-3, 4, 9, 10-tetracarboxy bisanhydride and bisimide. *Org. Lett.* **17**, 1882–1885, (2015).
3. Mahlmeister, B. *et al.* Helically Twisted Nanoribbons Based on Emissive Near-Infrared Responsive Quaterrylene Bisimides. *J. Am. Chem. Soc.* **144**, 10507–10514, (2022).
4. Ouyang, G. *et al.* Intramolecular Energy and Solvent-Dependent Chirality Transfer within a BINOL-Perylene Hetero-Cyclophane. *Angew. Chem. Int. Ed.* **61**, e202206706, (2022).
5. Domingos, S. R., Martin, K., Avarvari, N., & Schnell, M. Water docking bias in [4] Helicene. *Angew. Chem. Int. Ed.* **58**, 11257–11261, (2019).
6. Maya, A. B. *et al.* Further naphthylcombretastatins. An investigation on the role of the naphthalene moiety. *J. Med. Chem.* **48**, 556-568, (2005).
7. Hernandez-Perez, A. C., Vlassova, A., & Collins, S. K. Toward a visible light mediated photocyclization: Cu-based sensitizers for the synthesis of [5] helicene. *Org. Lett.* **14**, 2988–2991, (2012).
8. Weh, M., Rühe, J., Herbert, B., Krause, A.-M. & Würthner, F. Deracemization of Carbohelicenes by a Chiral Perylene Bisimide Cyclophane Template Catalyst. *Angew. Chem. Int. Ed.* **60**, 15323–15327, (2021)
9. Khan, M. A. & Goss, D. J. Poly(A)-Binding Protein Increases the Binding Affinity and Kinetic Rates of Interaction of Viral Protein Linked to Genome with Translation Initiation Factors eIFiso4F and eIFiso4F·4B Complex. *Biochemistry* **51**, 1388–1395, (2012).
10. Yang, L.-P. *et al.* A supramolecular system that strictly follows the binding mechanism of conformational selection. *Nat. Commun.* **11**, 2740, (2020).
11. Rickhaus, M., Jundt, L. & Mayor, M. Determining Inversion Barriers in Atrop- isomers – A Tutorial for Organic Chemists. *CHIMIA* **70**, 192, (2016).
