# Supplementary material for: Preferential molecular recognition of heterochiral guests within a cyclophane receptor
Source: Nat Commun. 2023 Jan 16;14:243. doi: 10.1038/s41467-023-35851-3 (PMC9842753; doi:10.1038/s41467-023-35851-3)
Supplement: Supplementary file 3 — Description of Additional Supplementary Files [file 41467_2023_35851_MOESM3_ESM.pdf]

Description of Additional Supplementary Files

Supplementary Data 1: contains the cartesian coordinates of the calculated structures
